# S-Species-Stimulated Deep Reconstruction of Ultra-Homogeneous CuS Nanosheets for Efficient HMF Electrooxidation

**DOI:** 10.34133/research.0925

**Published:** 2025-11-14

**Authors:** Yongzhi Xiong, Mengyuan Qiu, Yihan Wang, Qi Liu, Dong Ouyang, Yajun Liu, Changzhou Chen, Jianchun Jiang, Mengmeng Fan, Kui Wang

**Affiliations:** ^1^ Institute of Advanced Carbon Conversion Technology, Fujian Provincial Key Laboratory of Biomass Low-Carbon Conversion, Huaqiao University, Xiamen, Fujian 361021, China.; ^2^ Key Lab of Biomass Energy and Material of Jiangsu Province, Institute of Chemical Industry of Forest Products, Chinese Academy of Forestry, Nanjing, Jiangsu 210042, China.; ^3^ College of Chemical Engineering, Nanjing Forestry University, Nanjing, Jiangsu 210042, China.

## Abstract

Efficient utilization of renewable biomass resources is one of the feasible approaches to address the massive consumption of fossil fuels accompanying severe resource crises and environmental pollution. Currently, 2,5-furandicarboxylic acid derived from the oxidation of biomass-based 5-hydroxymethylfurfural (HMF) is a valuable chemical as the alternative to the fossil resource-derived terephthalic acid. However, the development of high-performance and low-cost Cu-based electrocatalysts for the efficient HMF oxidation reaction (HMFOR) remains an enormous challenge. Guided by our theoretical prediction, we proposed a coordination-pyrolysis strategy to fabricate highly dispersed copper sulfide (CuS) nanosheets supported on N-doped porous carbon precatalyst (CuS@NC). The covalent S species trigger the deep reconstruction of CuS nanosheets, and the in situ generated SO_4_^2−^ not only promotes the formation of Cu^2+δ^ species but also facilitates the cleavage of α–C–H and –O–H bonds in HMF. The optimized CuS@NC achieved a high current density of 335 mA cm^−2^ at 1.50 V vs. reversible hydrogen electrode, representing a remarkable 628% enhancement over the control catalyst. This study integrates theoretical predictions with experimental investigations to systematically elucidate how S species promote the deep reconstruction of CuS nanosheets to enhance the HMFOR performance and proposes a scalable strategy for preparing ultra-uniform transition metal sulfide precatalysts.

## Introduction

Green biomass energy conversion is regarded as one of the most effective solutions to obtain clean energy and products [1–4]. In particular, 5-hydroxymethylfurfural (HMF) is one of the most promising biomass platform compounds, bridging biomass resources and the petrochemical industry through its conversion into numerous value-added 2,5-furandicarboxylic acid (FDCA). FDCA has been identified as one of the 12 priority compounds for establishing the “green” chemical industry owing to its broad application prospects in bio-based plastics, fine chemicals, and the pharmaceutical industry [5–8]. Conventional thermal-driven methods for converting HMF to FDCA suffer from the drawback of high temperatures, high oxygen pressures, the cost of noble metal catalyst [8–10]. Compared with the traditional thermocatalysis, electrocatalysis possesses more advantages in mild reaction conditions, broad substrate tolerance, and high operating safety, which shows great edge in the selective oxidation by employing a directional flow of electrons as an energy injection mode to drive the oxidation process [8,11,12]. Compared to the kinetically sluggish oxygen evolution reaction (OER), the HMF oxidation reaction (HMFOR) operates at a lower theoretical potential. The low potential enables the production of high-value-added FDCA at the anode and hydrogen evolution at the cathode, thereby reducing the overall energy consumption [13–15].

To date, a large number of HMFOR electrocatalysts have been designed, with Ni-based catalysts being the most widely studied due to their excellent electrooxidation activity. The excessively high oxidation activity of Ni-based catalysts leads to an increase in the OER activity, which reduces the Faradaic efficiency of HMFOR. Hence, the selection of reaction sites with moderate oxidative activity to balance oxidation kinetics between the electrode and HMF is crucial for achieving high efficiency in HMF electrooxidation. Compared to Ni-based materials, Cu-based catalysts represent more favorable and ideal candidates for high-efficiency HMF electrooxidation (HMFOR), owing to their inherently lower activity toward the 4-electron OER [16,17]. However, most reported Cu-based catalysts exhibited suboptimal HMFOR activity due to the lack of efficient design for oxidative sites. For instance, the Ce-doped Cu-mesh catalyst exhibited a current density of less than 20 mA cm^−2^ at 1.5 V vs. reversible hydrogen electrode (RHE) [18]. The Co_9_S_8_-Ni_3_S_2_/Cu electrode showed a current density of only 90 mA cm^−2^ at 1.44 V vs. RHE [19]. Recently, an effective strategy was designed by anchoring nucleophilic PO_4_^3−^ on M-OH to accelerate the nonelectrochemical oxidation of HMF [20], which offers a feasible approach to overcoming the limited HMFOR activity of Cu-based catalysts at low potentials. However, this strategy faces challenges in terms of uniformity and stability for constructing PO_4_^3−^/M-OH sites. The introduction of nonmetallic heteroatoms into transition metal-based materials appear as an effective strategy for optimizing electronic structures and enhancing catalytic performance [21–23]. Remarkably, sulfur species (S) doped in the Ni_3_S_2_ lattice can be reconstructed into sulfate ions (SO_4_^2−^) to form SO_4_^2−^|Ni(OH)_2_ sites by promoting efficient dehydrogenation of Ni–OH leading to the improved HMFOR performance [24]. This finding suggests that introducing S species into the lattice of transition metal catalysts enables in situ reconstruction to form SO_4_^2−^|M–OH sites (where M represents a transition metal), thereby promoting HMFOR. The construction of SO_4_^2−^|Ni(OH)_2_ sites via the in situ reconstruction of transition metal sulfides is expected to address challenges in uniformity and stability due to the structural advantages of regular distribution of heteroatoms and the strong interaction between heteroatoms and transition metals. Currently, the predominant approach to synthesizing transition metal sulfide electrodes is hydrothermal growth, which requires harsh reaction conditions and poses difficulties in growth control [25,26], and the transition metal sulfide electrodes still suffer from drawbacks of active sites agglomeration, poor electrical conductivity.

Based on the above discussion, we designed a precatalyst comprising highly dispersed copper sulfide (CuS) nanosheets supported on nitrogen-doped porous carbon (CuS@NC) via a coordination-pyrolysis strategy, and successfully constructed efficient HMFOR sites of SO_4_^2−^|Cu(OH)_2_ through its in situ reconstruction. Ab initio molecular dynamics (AIMD) simulations indicate the feasibility of reconstructing CuS to SO_4_^2−^|Cu(OH)_2_ sites. Density functional theory (DFT) calculations reveal that SO_4_^2−^ facilitates the charge transfer between the catalyst and HMF, thereby accelerating the nonelectrochemical oxidation of HMF. Furthermore, quasi-in situ x-ray diffraction (XRD), in situ Raman, and in situ electrochemical impedance spectroscopy (EIS) demonstrate the crucial role of S species in promoting the deep reconstruction of the CuS@NC electrode and improving the utilization efficiency of active sites in HMFOR.

## Results and Discussion

### Catalyst design

**Fig. 1.**
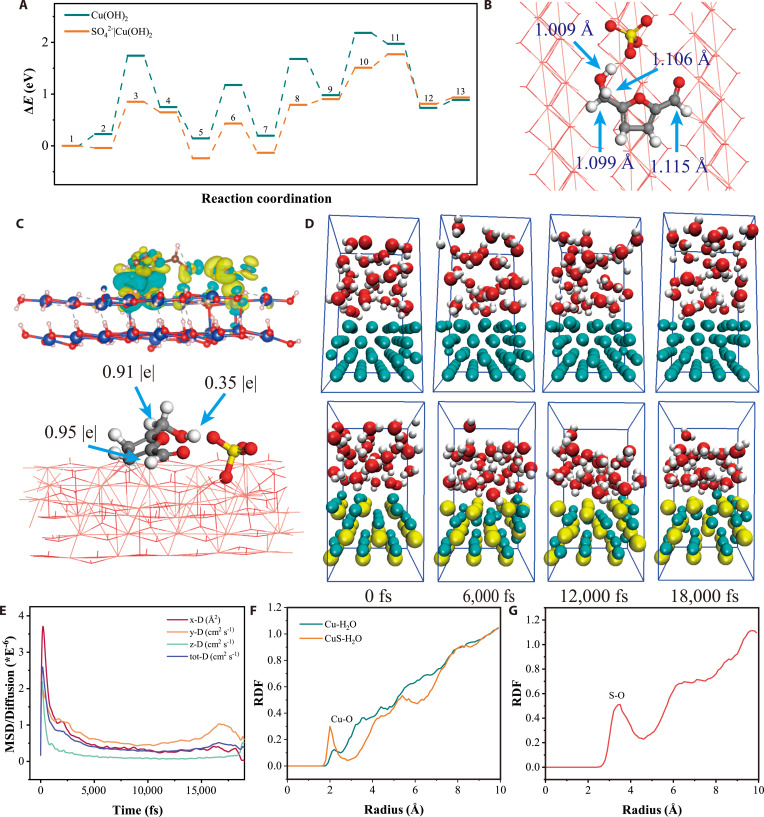
Theoretical simulation of catalysts (A to G). Reaction energy profiles for HMFOR at the Cu(OH)_2_ and SO_4_^2−^|Cu(OH)_2_ (A). Influence of SO_4_^2−^ for the α–C–H and –O–H (B). Differential charge density and Bader charge of HMF adsorbed on SO_4_^2−^|Cu(OH)_2_ (C). AIMD simulation snapshot (D). Mean square displacement (MSD)/diffusion coefficient of CuS-H_2_O (E). Radial distribution function (RDF) (Cu–O) (F) and RDF (S–O) (G).

To predict the influence of SO_4_^2−^ species incorporation on Cu(OH)_2_ during the HMFOR, we constructed Cu(OH)_2_ and SO_4_^2−^|Cu(OH)_2_ models including the electrochemical oxidation of the electrode and the nonelectrochemical oxidation of HMF. As shown in Fig. 1A, Fig. S1, and Table S1, the rate-limiting step reaction energy for HMFOR on SO_4_^2−^|Cu(OH)_2_ was 0.93 eV, while on Cu(OH)_2_, it was 1.51 eV. The decreased energy barrier for the SO_4_^2−^|Cu(OH)_2_ demonstrates that the modification of SO_4_^2−^ on the surface of Cu(OH)_2_ can effectively facilitate HMFOR. The dehydrogenation energy barrier of Cu–O–H decreased by an average of 0.52 eV, suggesting that SO_4_^2−^ species promotes the formation of highly active Cu^2+δ^ sites. On the other hand, the adsorption energy of HMF on Cu(OH)_2_ and SO_4_^2−^|Cu(OH)_2_ was 0.23 and −0.04 eV, respectively, indicating that the presence of SO_4_^2−^ species also considerably influences the adsorption of HMF, which properly affect the reaction kinetics of HMFOR. The influence of the SO_4_^2−^|Cu(OH)_2_ site on the reduction in bond order of α–C–H or –O–H bonds was illustrated in Fig. 1B and Fig. S2. After HMF adsorption on the surface of SO_4_^2−^|Cu(OH)_2_, the α–C–H and –O–H bonds adjacent to SO_4_^2−^ were elongated. For instance, the –O–H bond at the SO_4_^2−^|Cu(OH)_2_ site stretched from 0.975 to 1.009 Å compared to that on the Cu(OH)_2_. After adjusting the relative positions of SO_4_^2−^ and HMF, the elongation of the α–C–H and –O–H bonds adjacent to SO_4_^2−^ was attributed to the nucleophilic SO_4_^2−^ on the Cu(OH)_2_, which can facilitate the charge transfer between HMF and the catalyst. This perspective is also supported by differential charge density and Bader charge analyses (Fig. 1C, Fig. S3, and Table S2). The differential charge density revealed a marked enhancement in charge transfer when HMF was adsorbed on the SO_4_^2−^|Cu(OH)_2_ site. Further Bader charge analysis showed that the charge transfer of the H atoms in the α–C–H bonds and –O–H groups adjacent to SO_4_^2−^ increased from −0.03 |e|, −0.07 |e|, and −0.61 |e| to −0.05 |e|, −0.09 |e|, and −0.65 |e|, respectively. Therefore, the SO_4_^2−^|Cu(OH)_2_ site activates both the α–C–H bonds and the –O–H groups of HMF by enhancing charge transfer.

Given the poor robustness associated with the stepwise anchoring of SO_4_^2−^ and the reconstruction of transition metal catalysts in alkaline electrolytes, the in situ anchoring of SO_4_^2−^ during the electrocatalyst reconstruction process is a more promising strategy for constructing SO_4_^2−^|Cu(OH)_2_ sites at the electrode [20]. To evaluate the feasibility of embedding S species into Cu-based catalysts for promoting both their oxidative reconstruction and the in situ formation of SO_4_^2−^, a periodic solid–liquid interface model of Cu-H_2_O and CuS-H_2_O was constructed for AIMD simulations. The visual representations of the structures of the Cu-H_2_O and CuS-H_2_O solid–liquid interface models at different simulation times are provided in Fig. 1D and Movies S1 and S2. The larger mean square displacement (MSD) of the CuS-H_2_O solid–liquid interface model during the AIMD simulation confirms that the embedding of S species enhances the reconstruction of CuS to Cu(OH)_2_ (Fig. 1E and Fig. S4). To clearly illustrate this enhanced oxidation reconstruction, the radial distribution functions (RDFs) of the Cu-H_2_O and CuS-H_2_O models after simulation were calculated (Fig. 1F and G). The results indicate that the embedding of S species in the CuS lattice substantially increased the coordination number of Cu–O bonds in CuS-H_2_O compared to that in Cu-H_2_O, suggesting that the reconstructed CuS exhibits stronger oxidation activity. In addition, the extra S–O bonds formed in CuS-H_2_O simulation indicated that the CuS not only generates copper hydroxide but also produces SO_4_^2−^ species. Based on the above discussion, the introduction of S species into Cu-based catalysts is theoretically expected to greatly improve HMFOR performance as it markedly enhanced the reconstruction of Cu-based catalysts and reduced the energy barrier for the oxidation of HMF.

### Design and characterization of catalysts

**Fig. 2.**
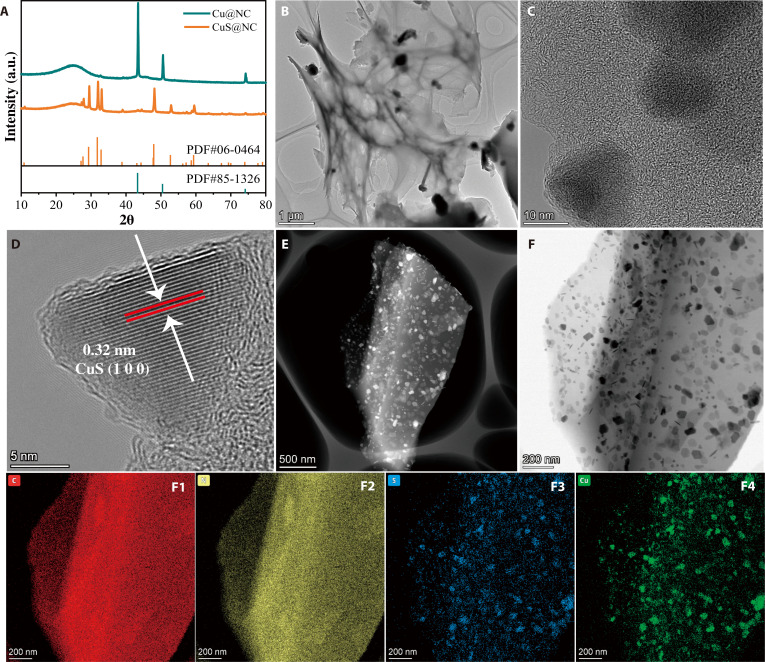
Characterization of the crystal structure and morphology of precatalysts (A to F). XRD patterns of Cu@NC and CuS@NC (A). TEM (B and C), HR-TEM (D), HADDF-STEM (E), and TEM-mapping (F) of CuS@NC [(F1) to (F4) show the mapping diagrams of the distribution of C, N, S, and Cu, respectively].

Based on the theoretical results, we successfully designed ultra-homogeneous CuS nanosheets supported by a 3-dimensional interconnected porous carbon framework precatalyst (CuS@NC) using a metal-chitosan coordination strategy (Scheme S1) through the formation of the Cu^2+^ hydrogel through coordination between the amino functional groups of chitosan and Cu^2+^ ions and then under high-temperature pyrolysis. The control catalyst of Cu nanoparticles supported by the 3-dimensional interconnected porous carbon framework (Cu@NC) was fabricated. The diffraction peaks of CuS@NC and Cu@NC were indexed to hexagonal-phase CuS (PDF #06-0464) and cubic-phase Cu (PDF #85-1326), respectively, in XRD patterns (Fig. 2A), indicating that S species are embedded into the lattice of Cu nanoparticles through the hydrothermal sulfidation process, thereby transforming Cu nanoparticles into CuS nanosheets. The CuS@NC maintained a 3-dimensional interconnected carbon framework structure, as observed via the scanning electron microscope and transmission electron microscope (TEM) images (Fig. 2B and C and Figs. S5, S6, and S7), indicating that the sulfidation process did not destroy the 3-dimensional interconnected structure. The well-developed and abundant 3-dimensional porous structure acts as a crucial mass transfer pathway in the HMFOR process, facilitating the transport of OH^−^ and the reaction substrate (HMF) during the electrocatalytic reaction. High-resolution TEM (HR-TEM) images in Fig. 2D and Fig. S8A showed CuS nanosheets with d-spacings of 0.32 nm, which closely matched the lattice fringes of the (100) planes of CuS [27]. In comparison, the d-spacing of Cu@NC before sulfidation was 0.18 nm (Fig. S8B), corresponding to the (200) plane of Cu [28–30]. The increased layer spacing can be attributed to lattice strain and the lattice expansion induced by the incorporation of S atoms into the lattice of Cu nanoparticles [29,30]. The corresponding TEM elemental mapping demonstrated that the distributions of Cu and S elements in CuS@NC exhibited a high degree of spatial correlation, with CuS dispersed uniformly within the carbon framework as ultra-homogeneous nanosheets (Fig. 2F).

**Fig. 3.**
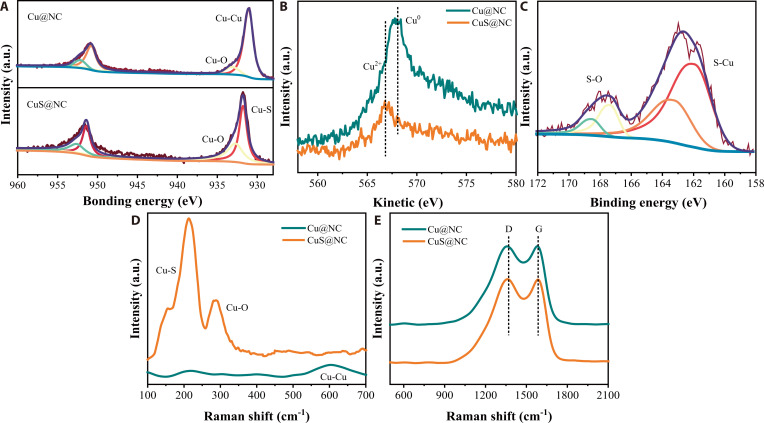
Spectral characterization of catalysts (A to E). High-resolution XPS spectra of Cu 2p (A) and Cu LMM Auger spectra (B) for Cu@NC and CuS@NC. S 2p spectra of CuS@NC (C). Raman spectra of Cu@NC and CuS@NC (D and E).

X-ray photoelectron spectroscopy (XPS) was conducted to investigate the chemical composition and coordination environment in Cu@NC and CuS@NC. The difference in elemental composition suggests the introduction of S in CuS@NC (Fig. S9). All the samples displayed C, N peaks, indicating the presence of N-doped carbon structure. The enhanced H_2_O adsorption energy of the N-doped carbon structure in DFT calculations demonstrates that N-doping facilitates improved hydrophilicity of the catalyst’s carbon support, thereby enhancing the accessibility of active sites during the reaction process (Fig. S10). High-resolution Cu 2p spectra and LMM spectra demonstrated that the Cu element in Cu@NC primarily existed as Cu^0^ in its metallic state, while the Cu in CuS@NC predominantly existed as Cu^2+^ in the form of Cu-S (Fig. 3A and B). The presence of S-Cu bonds was also observed in the high-resolution S 2p XPS spectra and Raman spectra (Fig. 3C and D). Additionally, both Cu@NC and CuS@NC exhibited the presence of Cu^2+^ in the form of Cu-O, which can be attributed to the Cu oxidation by H_2_O and O_2_ in the air [31,32].

### Validation for precatalysts’ reconstruction

Quasi-in situ XRD, in situ Raman spectroscopy, and in situ EIS were employed to investigate the reconstruction of electrodes. As shown in Fig. S11, the area of the cyclic voltammetry (CV) curves for both Cu@NC and CuS@NC electrodes initially expanded as the number of CV scans increased and gradually stabilized during the activation process. Notably, the CV curve area of the CuS@NC electrode was considerably larger than that of the Cu@NC electrode, indicating that the electrochemical activity of CuS@NC was higher than that of Cu@NC. Specifically, during the activation process, sulfur species (S^2−^) are oxidized to sulfate (SO_4_^2−^) while Cu^2+^ coordinated with hydroxide ions (OH^−^) from the electrolyte to form Cu(OH)_2_. The reaction process is as follows:

CuS → Cu^2+^ + S^2−^

S^2−^ +8OH^−^-8e^-^ → SO_4_^2−^+ 4H_2_O

Cu^2+^ + 2OH^−^ → Cu(OH)_2_

*x*Cu^2+^ + *y*SO_4_^2−^ + 2(*x*-*y*)OH^−^ → Cu*_x_*(SO_4_)*_y_*(OH)_2(*x*-*y*)_

**Fig. 4.**
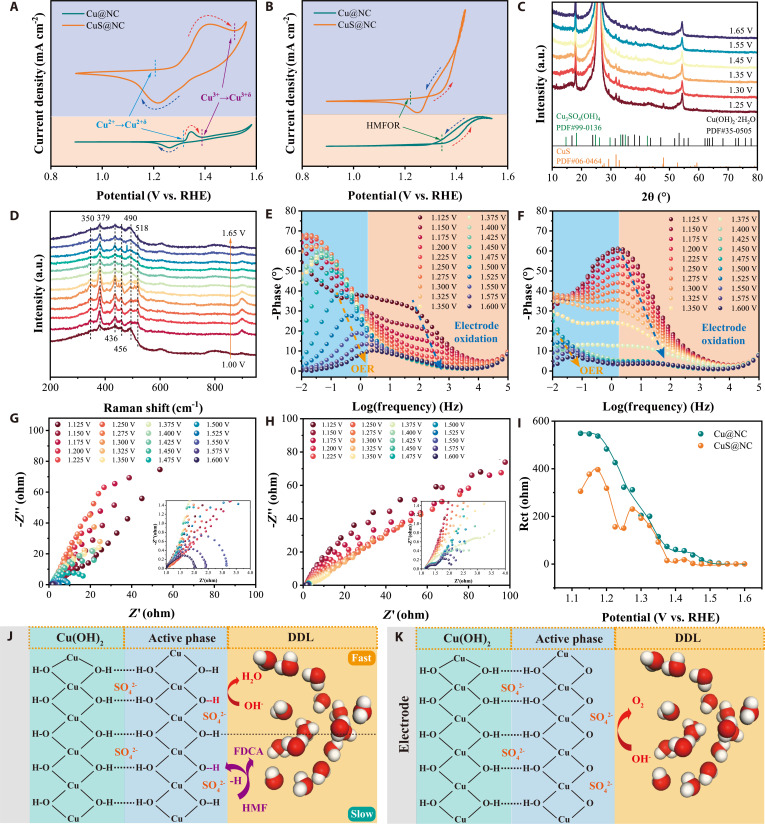
Verification of catalyst reconstruction (A to K). CV polarization curves of the reconstructed Cu@NC and CuS@NC electrodes in 1 M KOH (A) and 1 M KOH with 100 mM HMF (B). Quasi-in situ XRD patterns (C) and in situ Raman (D) spectra of CuS@NC. Bode phase plots of Cu@NC (E) and CuS@NC (F) in 1 M KOH. Nyquist plots of Cu@NC (G) and CuS@NC (H) in 1 M KOH. Resistance of the electrode interface reaction (R_1_) on Cu@NC and CuS@NC (I). Schematic illustrations of the HMFOR (J) and OER (K) system at the reconstructed CuS@NC electrode interface (SO_4_^2−^|Cu(OH)_2_).

To compare the oxidation activity of the reconstructed electrodes, the CV curves of the reconstructed Cu@NC and CuS@NC were systematically analyzed at a scan rate of 5 mV s^−1^. As illustrated in Fig. 4A, the CuS@NC electrode demonstrated notably lower oxidation potential for the transitions of Cu^2+^→Cu^2+δ^ (potential I) compared to the Cu@NC electrode, along with a marked enhancement of current density. This suggests that the incorporation of S species into CuS@NC markedly facilitates the deep reconstruction of the Cu^2+^ sites toward higher valence state Cu^2+δ^ sites with HMFOR activity, which is consistent with the predictions from DFT and AIMD simulations.

The higher potential II (Cu^3+^→Cu^3+^) of CuS@NC compared to Cu@NC can be attributed to the more prevalent oxidation of Cu^2+^ to Cu^2+δ^ (which, in turn, delays the conversion of Cu^2+δ^ to Cu^3+δ^)—a phenomenon driven by the lattice strain induced by the incorporation of S atoms. This strain promotes the deep reconstruction of CuS@NC, thereby increasing the number of highly active Cu^2+δ^ sites at the electrode/electrolyte interface. In contrast, due to the limited conversion of Cu^2+^ to Cu^2+δ^, the Cu@NC tends to preferentially convert Cu^2+δ^ species to Cu^3+δ^ as the potential increased. The CV curves obtained in 1 M KOH with 100 mM HMF electrolyte confirmed that the substantial decrease in potential I of CuS@NC reduced the reaction potential for the HMFOR. Additionally, benefiting from the deep reconstruction stimulated by the incorporation of S species, the HMFOR current density of CuS@NC was markedly increased (Fig. 4B).

To directly demonstrate that the incorporation of S species into CuS lattice triggers its deep reconstruction, a series of in situ characterizations were performed. The quasi-in situ XRD patterns recorded at different potentials revealed phase transformation behaviors of the 2 catalysts (Fig. S12A and Fig. 4C). Specifically, the results of quasi-in situ XRD tests showed notable differences in the degree of reconstruction between the 2 electrodes, despite the fact that both the reconstructed Cu@NC and CuS@NC electrodes exhibited diffraction peaks corresponding to new Cu-hydroxides. The Cu@NC electrode retained distinct diffraction peaks of metallic phase Cu (PDF #85-1326) at all potentials, and there was no decreasing trend with the increase of potential. In contrast, the characteristic diffraction peaks of CuS (PDF #06-0464) in CuS@NC electrode gradually diminished with increasing potential, with the emergence of new peaks corresponding to Cu_3_SO_4_(OH)_4_ (PDF #99-0316) and Cu(OH)_2_·2H_2_O (PDF #35-0505), indicating that CuS@NC undergoes a more thorough reconstruction than Cu@NC.

In situ Raman was also employed to gain deeper insights into the reconstruction and phase transition at the electrode|electrolyte interface. As shown in Fig. 4D and Fig. S12B, the peak near 490 cm^−1^, attributed to Cu(OH)_2_, was observed at all potentials for both Cu@NC and CuS@NC after reconstruction [33]. For the CuS@NC, the peak corresponding to the B_1g_(Cu-O) vibration in the range of 350 to 380 cm^−1^ appeared at potentials ≥ 1.10 V vs. RHE, whereas for Cu@NC, the corresponding peak did not appear until the potential above 1.45 V vs. RHE [34]. This indicates that the CuS@NC electrode undergoes more pronounced oxidation reconstruction at lower potential by dehydrogenation of Cu–O–H compared to Cu@NC. On the other hand, the peaks at 436 and 456 cm^−1^ of the CuS@NC electrode corresponded to SO_3_^2−^ or SO_4_^2−^, due to the overlap between the SO_4_^2−^ *ν*_2_ mode and the SO_3_^2−^ *ν*_4_ (E) bending mode [35,36]. The S 2p XPS signals attributed to SO_4_^2−^ of the reconstructed CuS@NC electrode (Fig. S13) confirmed that the Raman signals at 436 and 456 cm^−1^ were ascribed to SO_4_^2−^. Furthermore, the changes in the relative intensity of the peaks at 490 and 518 cm^−1^ in CuS@NC with increasing potential can be attributed to the influence of in situ generated SO_4_^2−^ on the deprotonation behavior of Cu(OH)_2_ species. The quasi-in situ XRD and in situ Raman spectroscopy analyses illustrate that CuS@NC undergoes a more thorough reconstruction than Cu@NC, and this enhanced reconstruction is attributed to the introduction of S species.

In situ EIS spectra were utilized to further corroborate the findings obtained from quasi-in situ XRD and in situ Raman spectroscopy. As depicted in Fig. 4E and F, the in situ Bode spectra of Cu@NC and CuS@NC in 1 M KOH revealed their reconstruction processes between the electrodes and inner Helmholtz layer [13,37]. The electro-oxidative dehydrogenation of Cu(OH)_2_ or SO_4_^2−^|Cu(OH)_2_ sites and OER were correspondingly reflected in the high-frequency region (the oxidation of the inner of electrode) and the low-frequency region (the heterogeneous charge distribution induced by OER), respectively. For Cu@NC, the Cu^2+^(OH)_2_ species can be electrochemically oxidized to Cu^2+δ^O_2_H_2-δ_ at potentials above 1.350 V vs. RHE, whereas 1.250 V vs. RHE was sufficient for the electro-oxidation of SO_4_^2−^|Cu^2+^(OH)_2_ to SO_4_^2−^|Cu^2+δ^O_2_H_2-δ_. This implied that the SO_4_^2−^|Cu^2+δ^O_2_H_2-δ_ species exhibits enhanced performance for the electro-oxidation of Cu^2+^→Cu^2+δ^. On the other hand, the potential for OER observed on CuS@NC is higher compared to that on Cu@NC, which further validates the conclusion drawn from in situ Raman spectroscopy and CV measurements that the more prevalent conversion of Cu^2+^ into Cu^2+δ^ species in the CuS@NC delays the onset of the OER. Further analysis of the in situ Bode plots for Cu@NC and CuS@NC after the addition of HMF revealed that the HMFOR potential was found to be highly correlated with the potential of the Cu^2+^ → Cu^2+δ^ transition in the KOH electrolyte and the potential corresponding to the HMFOR for CuS@NC is notably lower compared to that of Cu@NC, in agreement with the CV results (Fig. 4A and B and Fig. S14). The in situ Nyquist plots (Fig. 4G and H) and their fitted charge transfer resistance (Fig. 4I) also demonstrated that the incorporation of S species into CuS lattice considerably reduces the charge transfer resistance (R_1_) for the oxidation dehydrogenation processes during the deep reconstruction, thereby facilitating the HMFOR. After the addition of HMF, the CuS@NC electrode exhibited a single charge-transfer controlled step, indicating its higher efficiency in oxidizing HMF (Fig. S15).

The quasi-in situ XRD, in situ Raman, and in situ EIS spectroscopy confirm the reasonableness of our AIMD and DFT prediction conclusion and demonstrate that the Cu@NC and CuS@NC electrodes, as pre-catalysts, are reconstructed into true active site Cu(OH)_2_ or SO_4_^2−^|Cu(OH)_2_ in KOH electrolyte. The S species embedded in the CuS@NC lattice not only facilitate the deep reconstruction of the electrode but also generate SO_4_^2−^ to form localized SO_4_^2−^|Cu(OH)_2_ sites at the electrode|electrolyte interface. As illustrated in Fig. 4J to K and Fig. S16, the reconstructed SO_4_^2−^|Cu(OH)_2_ species facilitated the oxidation of Cu^2+^ to Cu^2+δ^ and concurrently accelerated the reduction of Cu^2+δ^ by HMF, thereby delaying the emergence of Cu^3+δ^ species with OER activity. This mechanism enables a more profound coupling between electrode oxidation and the nonelectrochemical oxidation of HMF, markedly enhancing the HMFOR activity of the CuS@NC electrode.

**Fig. 5.**
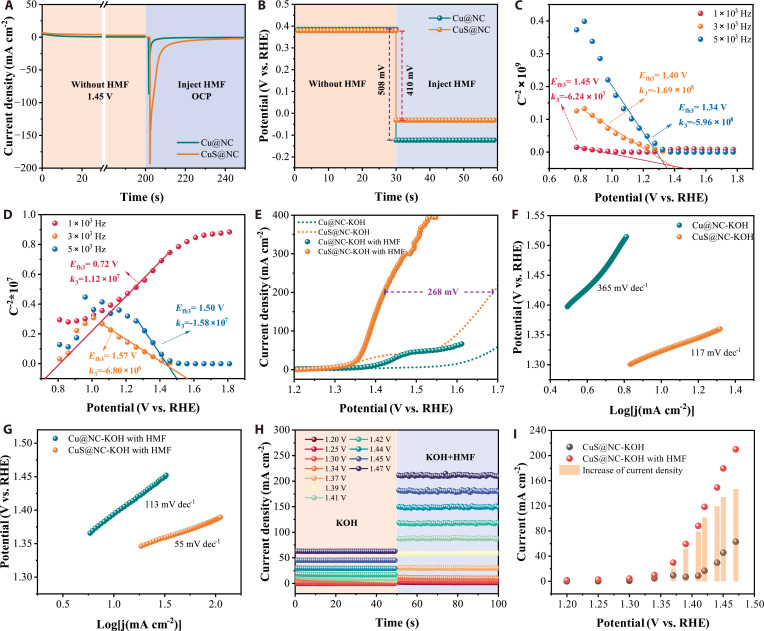
HMFOR performance testing (A to I). Periodic electrochemical measurements (A) and OCP (B) of Cu@NC and CuS@NC. Mott Schottky curves of Cu@NC (C) and CuS@NC (D). LSV polarization curves (E). Tafel plots measured in KOH (F) and KOH with HMF (G). *I*–*t* curve of CuS@NC (H). Comparison of current densities extracted from *i*–*t* curves at different applied potentials (I).

On the other hand, the role of SO_4_^2−^|Cu(OH)_2_ sites in facilitating the reduction of bond order in the α–C–H and –O–H bonds of HMF (as revealed by DFT calculations) was investigated through stepwise electrode oxidation experiments and open-circuit potential (OCP) measurements (Fig. 5A and B). The electrode was firstly electrochemically oxidized at the potential of 1.45 V vs. RHE in KOH electrolyte for 200 s, followed by the injection of HMF and setting the potential to open circuit potential. The current recorded under open-circuit conditions was attributed to the nonelectrochemical oxidation of α–C–H and –O–H in HMF by the oxidative species accumulated on the electrode surface, where the current intensity reflected the number of effective active sites, and the slope of the current decay indicated the reaction rate of these sites [1,20]. As shown in Fig. 5A, the CuS@NC with constructed SO_4_^2−^|Cu(OH)_2_ sites exhibited a considerably increased current density and a high reaction rate in the initial stage, which was attributed to the synergistic effect of the S species promoting the deep reconstruction of the CuS@NC electrode and the activation of the α–C–H and –O–H bonds in HMF by the SO_4_^2−^|Cu(OH)_2_ sites [20]. Notably, as the reaction time scale expanded, the reaction rate of the SO_4_^2−^|Cu(OH)_2_ sites decreased, likely due to the steric hindrance effect of the quasi-static electrode surface under open-circuit conditions, which limits the mass transfer of HMF. To validate this hypothesis, OCP tests were conducted (Fig. 5B). The results showed that the OCP drop of the CuS@NC electrode after the addition of HMF was smaller than that of Cu@NC, confirming that the steric hindrance effect of the SO_4_^2−^|Cu(OH)_2_ sites under quasi-static conditions restricted the mass transfer of HMF.

The Mott–Schottky plots, as shown in Fig. 5C and D, revealed that the linearity of the CuS@NC curve was reduced compared to that of Cu@NC, reflecting that the constructed SO_4_^2−^|Cu(OH)_2_ sites disrupted the uniformity of the electrode|electrolyte interface. This also reflects the influence of anchored SO_4_^2−^ on interfacial mass transfer. The calculated flat band potential (*E*_fb_) and carrier density (Na) demonstrated that CuS@NC exhibited a notable increase in both Na and *E*_fb_, indicating its enhanced electrooxidation activity (Table S3).

### HMFOR performance

The HMFOR performance of the reconstructed Cu@NC and CuS@NC was evaluated using a standard 3-electrode system in 1 M KOH electrolyte with 100 mM HMF, following activation by CV. Figure 5E shows the linear sweep voltammetry (LSV) curves of Cu@NC and CuS@NC with and without 100 mM HMF. The Cu@NC electrode had only a poor HMFOR activity with a current density of 46 mA cm^−2^ at a potential of 1.50 V vs. RHE. In comparison, the CuS@NC electrode achieved a current density of 335 mA cm^−2^ at the same potential, representing a 628% improvement over the Cu@NC electrode. On the other hand, the CuS@NC electrode exhibited exceptional competitive activity for the HMFOR, requiring only the potential of 1.42 V vs. RHE to achieve a high current density of 200 mA cm^−2^, which was 268 mV lower than that of the OER.

The fitted Tafel slopes in 1 M KOH without HMF (Fig. 5F) and with HMF (Fig. 5G) indicated that the incorporation of S species in CuS@NC substantially enhanced the HMFOR performance. According to previous studies, the potential–dynamic LSV curves cannot show the true current density of the HMFOR due to the influence of competing OER as well as the capacitive currents [16]. In order to obtain actual current densities closer to the HMFOR contribution, segmented *i*–*t* chronoamperometry was carried out, and the increment of current density after addition of HMF into electrolyte was attributed to the HMFOR (Fig. 5H and I and Figs. S17 and S18). The segmented *i*–*t* chronoamperometry demonstrated that the CuS@NC electrode exhibited markedly superior HMFOR performance compared to Cu@NC. At a potential of 1.47 V vs. RHE, the steady-state current density of CuS@NC increased by more than 6 times compared to Cu@NC. The CuS@NC not only exhibited considerably higher current density in KOH with HMF electrolyte but also demonstrated a much greater current value of *I*_KOH_ − *I*_KOH+HMF_, which reflects the true current density increment contributed by HMF.

**Fig. 6.**
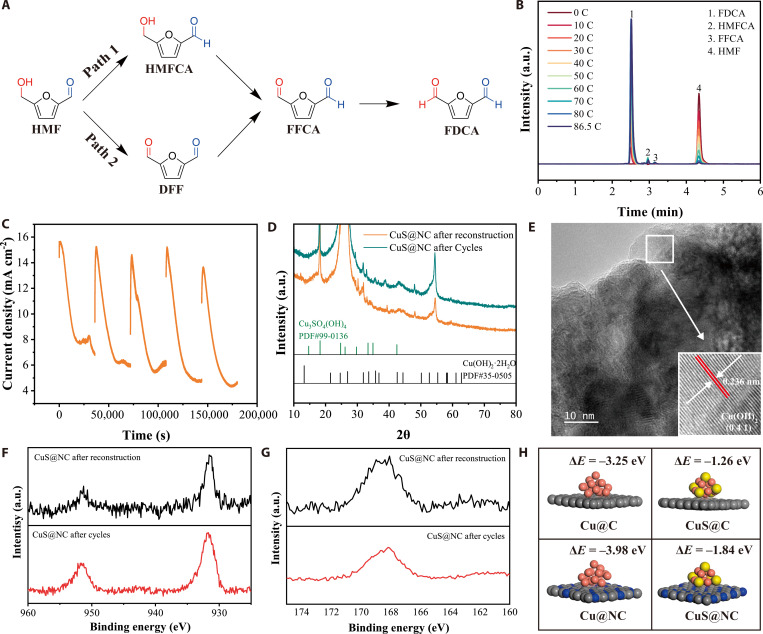
Reaction pathway and stability testing (A to H). Proposed reaction pathway for HMF oxidation (A). HPLC chromatogram of reaction products (B). Multi-cycle *i*–*t* test over 5 cycles (50 h) (C). XRD patterns of CuS@NC at different reaction stages (D). HR-TEM (E) of CuS@NC after cycling tests. High-resolution XPS spectra of Cu 2p (F) and S 2p (G) spectra of CuS@NC at different stages. Influence of N doping on catalyst stability (H).

The possible reaction pathways of HMFOR include the 5-hydroxymethyl-2-furoic acid (HMFCA) pathway (Path I) and the 2,5-furandicarboxaldehyde (DFF) pathway (Path II) (Fig. 6A). The high-performance liquid chromatography (HPLC) chromatogram was used to track the oxidation process of HMFOR at the potential corresponding to a current density of 10 mA cm^−2^ of CuS@NC in 1 M KOH with 10 mM HMF electrolyte. The oxidation of HMF on the CuS@NC electrode followed Path I (HMFCA), indicating that the aldehyde group of HMF is initially oxidized (Fig. 6B). Most literature report that the aldehyde group of HMF is more susceptible to oxidation in a strongly alkaline environment [2,38,39]. Our monitoring of the spontaneous oxidation of HMF in 1 M KOH electrolyte via HPLC also supported this conclusion, and HMF gradually oxidized to HMFCA in the strongly alkaline environment even without any applied potential (Fig. S19). Based on the HPLC spectra, the calculated HMF conversion rate was nearly 100%, while the FDCA selectivity and Faradaic efficiency were 99.3% and 98.4%, respectively.

To evaluate the stability of the CuS@NC electrode, we conducted 5 consecutive chronoamperometry (*i*–*t*) tests, with a cumulative duration of 50 h. After CV activation and each test, the electrode was thoroughly cleaned to prevent the products of the HMFOR from covering the active sites. After 5 cycles, the CuS@NC maintained a relatively high current density, indicating that it has excellent stability (Fig. 6C). The XRD analysis revealed that the cycled CuS@NC electrode preserved a nearly identical structure to its reconstructed state (Fig. 6D). Furthermore, the presence of the Cu(OH)_2_ (041) plane in the HR-TEM image and the widespread distribution of S and Cu elements (Fig. 6E and Fig. S20) strongly indicate the excellent preservation of SO_4_^2−^|Cu(OH)_2_ sites. The nearly identical Cu 2p and S 2p spectra in XPS characterization between the cycled electrode and the reconstructed electrode demonstrated the structure stability (Fig. 6F and G). The DFT calculations displayed that homogeneous N-doped not only optimized the mass transfer for HMFOR by improving hydrophilicity but also enhanced the stability of the catalyst by increasing the interaction between the support and CuS nanosheets (Fig. S10 and Fig. 6H) [40].

## Conclusion

In summary, DFT calculations confirmed that SO_4_^2−^ groups anchored on Cu(OH)_2_ lowered the energy barrier for generating Cu^2+δ^ species and activated α–C–H and –O–H in HMF. AIMD simulations further predicted the feasibility of incorporating S species in CuS@NC facilitating electrode deep reconstruction, with embedded S species transforming into SO_4_^2−^ anchored on Cu(OH)_2_. The CuS nanosheets supported on a 3-dimensional interconnected porous carbon pre-catalyst was successfully designed. A series of in situ characterizations demonstrated that the S species stimulated the deep reconstruction of the CuS@NC catalyst, promoting the formation of Cu^2+δ^ species with high HMFOR activity. Therefore, the CuS@NC electrode exhibited exceptional competitive activity for the HMFOR, requiring only a low potential of 1.42 V vs. RHE to achieve an ultrahigh current density of 200 mA cm^−2^, which was 268 mV lower than that required for OER. The CuS@NC achieved a high conversion rate of nearly 100%, a selectivity of 99.3%, and a Faradaic efficiency of 98.4%, and maintained a relatively high current density after 5 cycles. This study not only points out the crucial role of introducing S species to stimulate the deep reconstruction of Cu-based catalysts for effectively promoting HMFOR, but also provides a generalizable strategy for developing ultra-uniform transition metal-based nanosheet catalysts supported on porous carbon frameworks.

## Materials and Methods

### Materials

NH_3_·H_2_O (98%), chitosan (100 to 200 mPa⋅s), potassium hydroxide (KOH, 99.99%), ammonium formate (HPLC), DFF (98%), HMFCA (98%), and 2,5-furancarboxylic acid (FDCA, 98%) were procured from Shanghai McLean Biochemical Technology Co. Other chemicals, including Cu(NO_3_)_2_·3H_2_O (99%), HMF (99.0%), and FFCA (>98%), were purchased from Shanghai Aladdin Biochemical Technology Corporation. Thiourea (≥99%) was supplied by National Pharmaceutical Reagent Co. Nickel foam (NF) was obtained from ATTME-Technology-Corporation.

### Sample preparation

Firstly, 3 g chitosan (CS) was dissolved in 120 ml of CH_3_COOH solution (2%). Cu(NO_3_)_2_·3H_2_O (3 mM) was added and stirred thoroughly until the transparent and uniform blue gel-like solution was formed. Subsequently, the gel-like solution was transferred to a sealed container containing 80 ml of ammonia water and left to stand for 24 h. As the acetic acid in the solution was neutralized by the absorbed NH_3_, Cu^2+^ coordinated with the amino groups of chitosan to form a uniform Cu-CS hydrogel, and then the Cu-CS aerogel was obtained after freeze-drying. The Cu-CS aerogel was placed in a tube furnace and heated to 800 °C under an Ar atmosphere for 2 h with a heating rate of 5 °C min^−1^ to obtain the Cu nanoparticle supported by a 3-dimensional interconnected nitrogen-doped carbon catalyst (Cu@NC). Finally, the Cu@NC and 5 g of thiourea were dissolved in 70 ml of deionized water. The mixture was then transferred to a hydrothermal reactor and reacted at 120 °C for 12 h. The hydrothermal reaction product was collected, boiled, and washed thoroughly to obtain CuS nanosheets supported by a 3-dimensional interconnected porous carbon frameworks precatalyst (CuS@NC).

### Electrochemical measurements

All electrochemical measurements were conducted using a standard 3-electrode configuration. In situ EIS was performed on a BioLogic SP-200 electrochemical workstation. All other electrochemical characterizations were carried out using a CHI 660E electrochemical workstation. The 3-electrode system comprised a self-supported NF electrode as the working electrode, a graphite sheet as the counter electrode, and a Hg/HgO electrode as the reference electrode. The catalyst (3 mg) was thoroughly ground and completely dispersed in 100 μl of a 1:1 (v/v) ethanol/water solution, followed by the addition of 10 μl of Nafion D521 solution. After 30 min of ultrasonication, the homogeneous ink was precisely drop-cast onto an NF substrate (1 cm × 2 cm), forming a uniform coating area of ~1 cm^2^. Prior to electrochemical measurements, all working electrodes were activated by running 50 cycles of CV in the potential range of 0.69 to 1.59 V vs. RHE (without IR compensation) at a scan rate of 50 mV s^−1^ to reconstruct the pre-catalyst into the actual catalytically active species. All reported potentials were converted to the RHE scale using the following equation: *E*_RHE_ = *E*_HgO/Hg_ + 0.098 V + 0.0592 V × pH. All the LSV and *i*–*t* curves were scanned at a rate of 5 mV s^−1^ with 95% iR compensation. To evaluate the stability of the CuS@NC electrode, we conducted 5 consecutive chronoamperometry (*i*–*t*) tests, with a cumulative duration of 50 h, in an electrolyte consisting of 30 ml of 1 M KOH with 100 mM HMF. After CV activation or each test, the electrode was thoroughly cleaned to prevent the products of the HMFOR from covering the active sites.

### Product quantification

The oxidation pathway of HMF was investigated using chronoamperometry coupled with HPLC to determine the conversion rate, selectivity, and Faraday efficiency. During the electrochemical process in a 3-electrode system, 10 μl of electrolyte was periodically extracted after every 10 C charge input and transferred to 1 ml of ultrapure water. Subsequently, 10 μl of HCl was added to adjust the pH of the solution. Subsequently, the composition of the electrolyte was quantitatively analyzed by HPLC. The Agilent 1260 Infinity II HPLC system, equipped with a Shim-pack GWS 5 μm C18 column (4.6 mm × 250 mm) and a UV–Vis detector, was employed to detect the substrates, intermediates, and products of HMFOR at a column temperature of 35 °C and a detection wavelength of 265 nm. The HPLC analysis was performed using isocratic elution with a mobile phase comprising 70% solution A (5 mM ammonium formate) and 30% solution B (methanol). The separation of reactants, intermediates, and products was achieved at a flow rate of 1 ml min^−1^ for 6 min. The identification and quantification of electrolyte components were conducted based on the retention times of commercially available standard substances and their corresponding calibration curves. The conversion (%) of HMF, selectivity (%), and FE (%) were calculated using the following 3 equations:

$$\begin{array}{c} \text{Conversion}\;\left(\%\right)=\frac{\text{mole of substrate consumed HMF}}{\text{mole of initial HMF}}\times 100\% \end{array}$$ (1)

$$\begin{array}{c} \text{Selectivity}\;\left(\%\right)=\frac{\text{mole of product formed FDCA}}{\text{mole of initial substrate}\;}\times 100\% \end{array}$$ (2)

$$\begin{array}{c} \mathrm{FE}\;\left(\%\right)=\frac{\text{mole of product formed FDCA}}{\text{total charge passed}/\left(n*F\right)}\times 100\% \end{array}$$ (3)

where *n* is the number of electron transfers for each product, and *F* is the Faraday constant (96,485 C mol^−1^).

### Theoretical calculations

DFT and AIMD simulations were performed using the Vienna ab initio simulation package with the Perdew–Burke–Ernzerhof generalized gradient approximation [41,42]. The calculations employed a plane-wave basis set with a cutoff energy of 450 eV. A gamma-centered k-point mesh (1 × 1 × 1) was utilized for Brillouin zone integration. In the construction of the surface models of Cu(OH)_2_ and SO_4_^2−^|Cu(OH)_2_, a 2-layer Cu(OH)_2_ structure was retained. The unit cell size of the Cu(OH)_2_ model was set to 14.9 × 15.4 × 20.9 Å to avoid periodic interactions between HMF molecules. To investigate the influence of the SO_4_^2−^ on the α–C–H bond and –O–H group of HMF, 3 adsorption configurations of SO_4_^2−^-modified Cu(OH)_2_ were constructed with SO_4_^2−^ positioned at different sites. The convergence criteria were set to 10^−5^ eV for total energy of electronic iteration and 0.03 eV/Å for atomic forces of geometric structure relaxation. The adsorption energy of catalyst surface for reactant was defined by Eq. 4 as follows:

$$\Delta E=E_{\text{molecule}/\text{surface}}-\left(E_{\text{molecule}}+E_{\text{surface}}\right)$$ (4)

where *E*_molecule/surface_, *E*_molecule_, and *E*_surface_ are the total energy of the molecules adsorbed on the catalyst surface, molecules in the vacuum, and clean catalyst surface, respectively. Based on this definition, a more negative Δ*E* value indicates a more energetically favorable (exothermic) reaction.

## Data Availability

All data of this study are available from the corresponding authors upon request.

## Supplementary Materials

Supplementary 1 Figs. S1 to S20 Scheme S1 Tables S1 to S3 Movies S1 and S2
