# Supplementary material for: S-Species-Stimulated Deep Reconstruction of Ultra-Homogeneous CuS Nanosheets for Efficient HMF Electrooxidation
Source: Research (Wash D C). 2025 Nov 14;8:0925. doi: 10.34133/research.0925 (PMC12615154; doi:10.34133/research.0925)
Supplement: Supplementary 1 — Figs. S1 to S20 Scheme S1 Tables S1 to S3 Movies S1 and S2 [file research.0925.f1.zip › SUPPLEMENTARY MATERIALS_Figures S1 to S5.docx]

**S-species****-Stimulated Deep-reconstruction of Ultra-homogeneous CuS Nanosheets for Efficiently HMF Electrooxidation**

Yongzhi Xiong ^1^, Mengyuan Qiu ^1^, Yihan Wang ^1^, Qi Liu^1^, Dong Ouyang ^1^, Yajun Liu ^1^, Changzhou Chen ^1^, Jianchun Jiang ^1, 2, *^, Mengmeng Fan ^3,*^, Kui Wang ^1, 2, *^

*^1^ Institute of Advanced Carbon Conversion Technology, Fujian Provincial Key Laboratory of Biomass Low-Carbon Conversion, Huaqiao University, Xiamen, Fujian 361021, China*

*^2^ Key Lab. of Biomass Energy and Material of Jiangsu Province, Institute of Chemical Industry of Forest Products, Chinese Academy of Forestry, Nanjing, Jiangsu 210042, China*

*^3^ College of Chemical Engineering, Nanjing Forestry University, Nanjing, Jiangsu 210042, China*

Correspondence should be addressed to Jianchun Jiang, jiangjc@icifp.cn; Mengmeng Fan, fanmengmeng370@njfu.edu.cn and Kui Wang, wangkui@caf.ac.cn

**
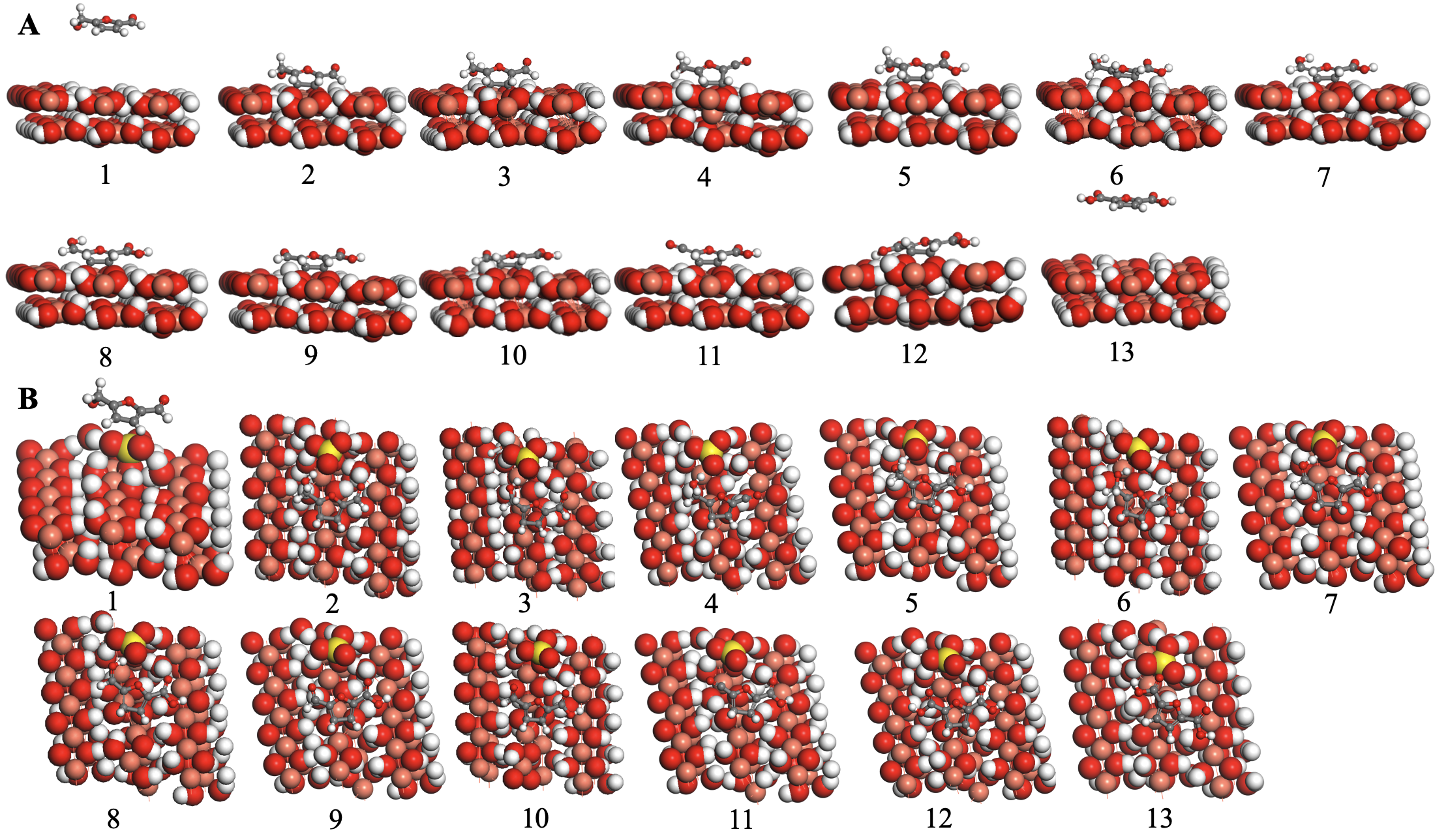
Fig. S1** Model structures of the HMFOR process on the Cu(OH)_2_ (A) SO_4_^2-^|Cu(OH)_2_ (B).


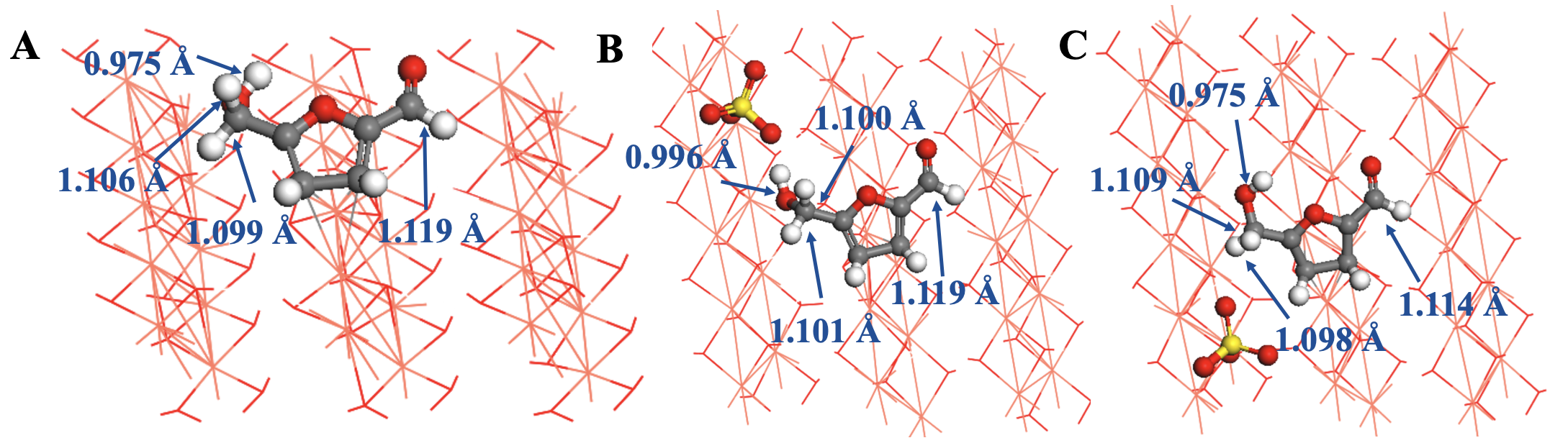


**Fig. S2** The bond lengths of α–C–H and –O–H after the adsorption of HMF molecules on the surfaces of Cu(OH)_2_ (A) and SO_4_^2-^|Cu(OH)_2_ (B and C).


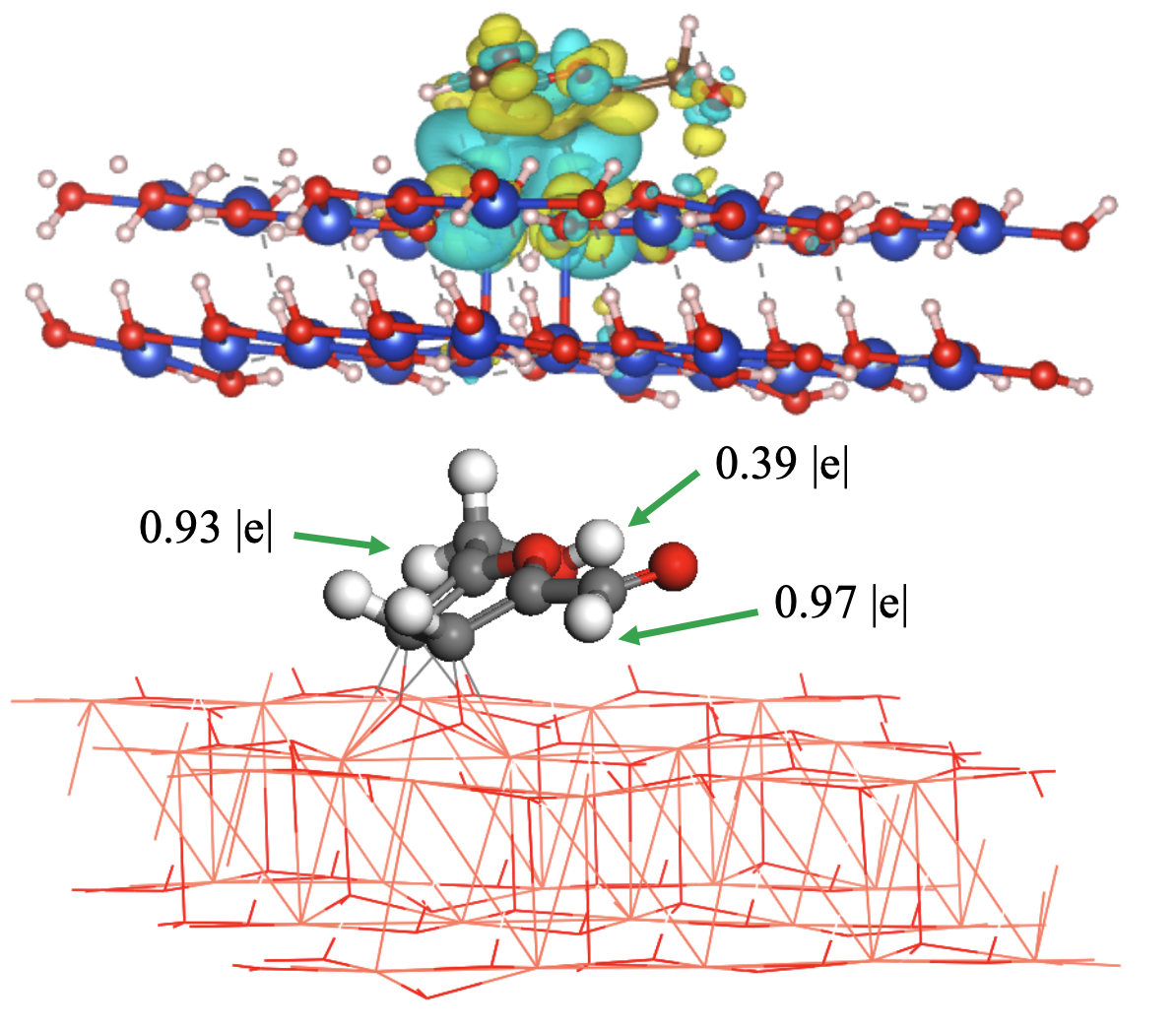


**Fig. S3** Differential charge density and Bader charge of HMF adsorbed on Cu(OH)_2_.


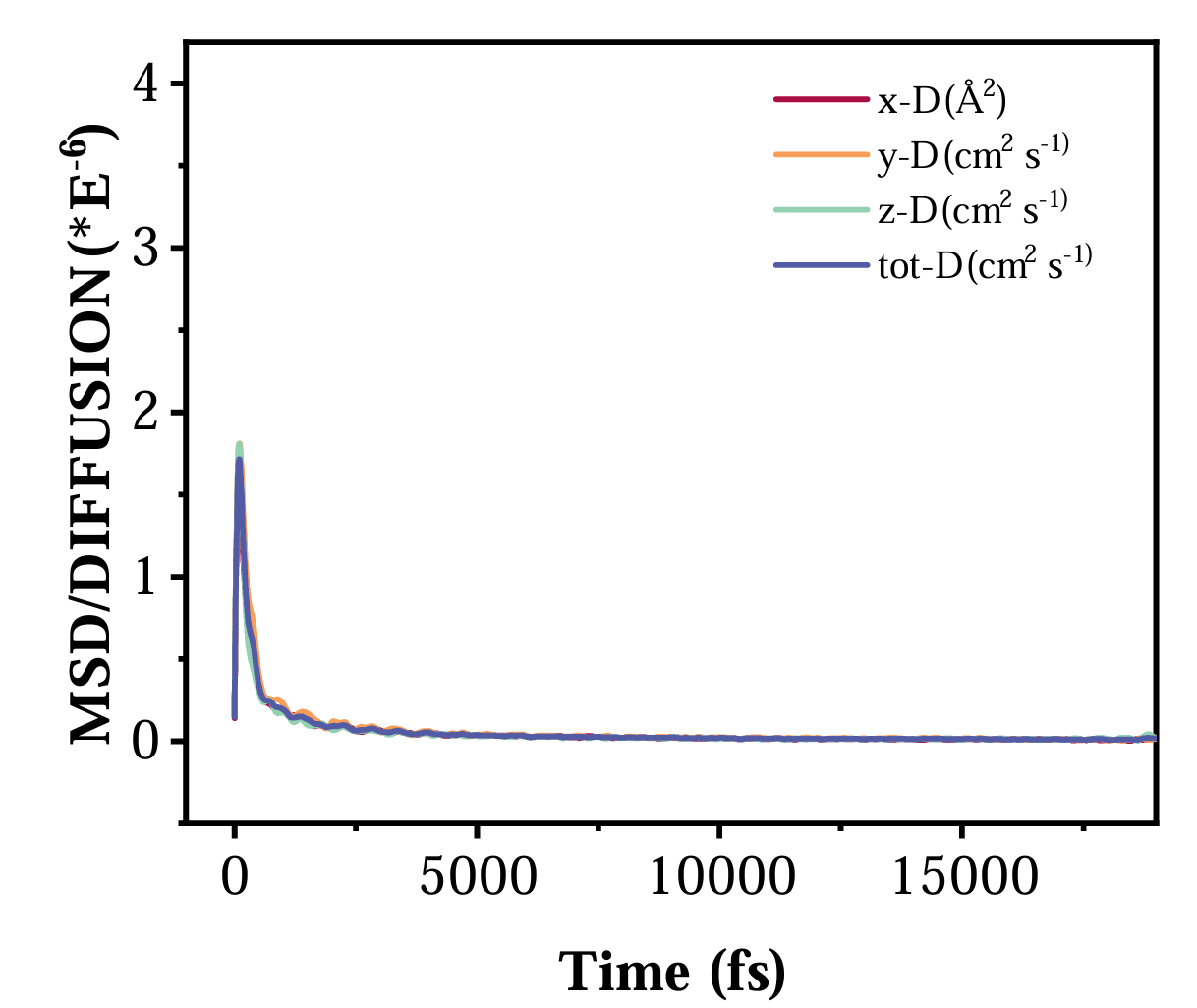


**Fig. S4** Mean square displacement (MSD)/diffusion coefficient of Cu-H_2_O.


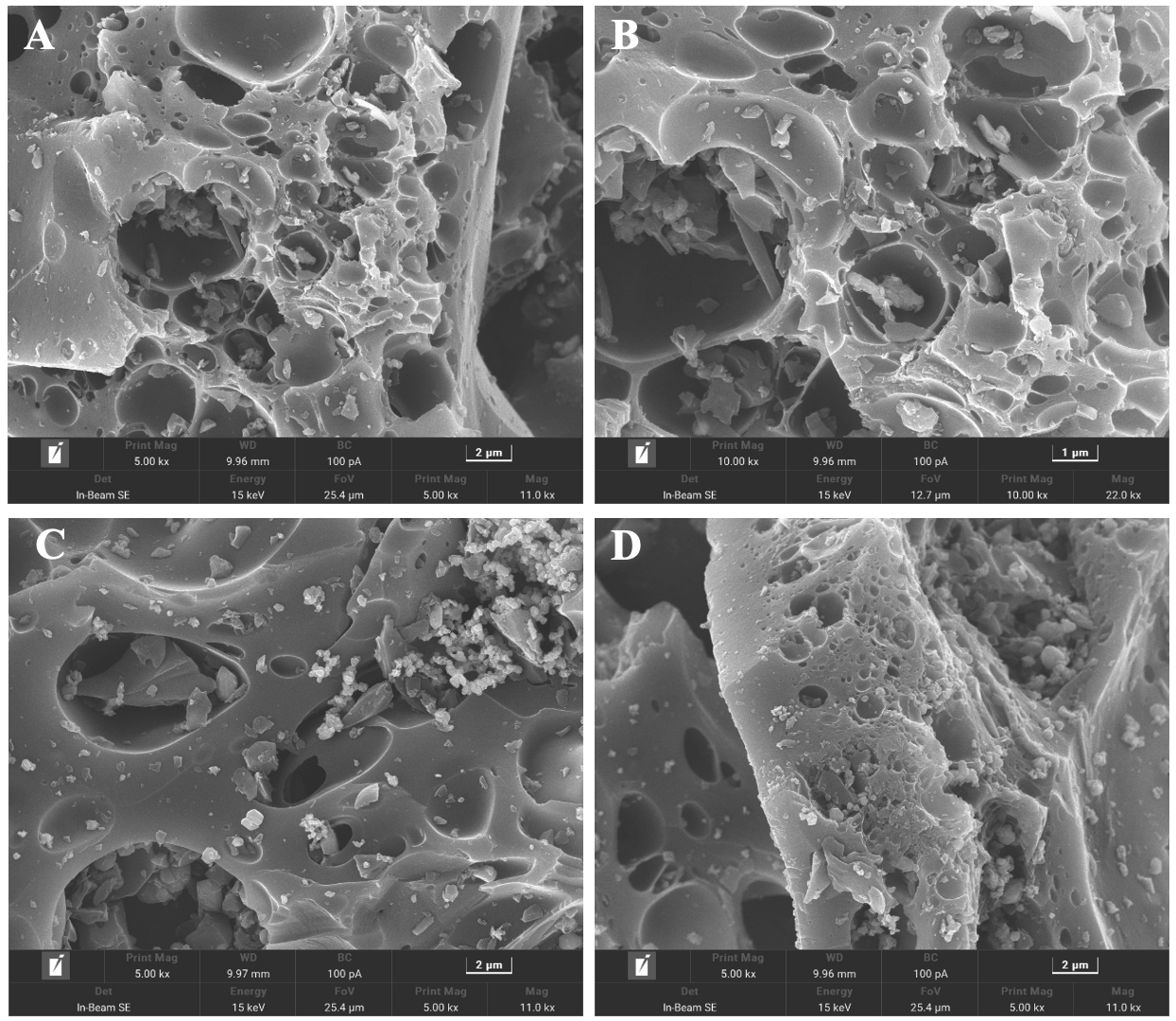


**Fig. S5** SEM of Cu@NC (A and B) and CuS@NC (C and D).
