# Supplementary material for: S-Species-Stimulated Deep Reconstruction of Ultra-Homogeneous CuS Nanosheets for Efficient HMF Electrooxidation
Source: Research (Wash D C). 2025 Nov 14;8:0925. doi: 10.34133/research.0925 (PMC12615154; doi:10.34133/research.0925)
Supplement: Supplementary 1 — Figs. S1 to S20 Scheme S1 Tables S1 to S3 Movies S1 and S2 [file research.0925.f1.zip › SUPPLEMENTARY MATERIALS_Figures S11-S20.docx]

**S-species****-Stimulated Deep-reconstruction of Ultra-homogeneous CuS Nanosheets for Efficiently HMF Electrooxidation**

Yongzhi Xiong ^1^, Mengyuan Qiu ^1^, Yihan Wang ^1^, Qi Liu^1^, Dong Ouyang ^1^, Yajun Liu ^1^, Changzhou Chen ^1^, Jianchun Jiang ^1, 2, *^, Mengmeng Fan ^3,*^, Kui Wang ^1, 2, *^

*^1^ Institute of Advanced Carbon Conversion Technology, Fujian Provincial Key Laboratory of Biomass Low-Carbon Conversion, Huaqiao University, Xiamen, Fujian 361021, China*

*^2^ Key Lab. of Biomass Energy and Material of Jiangsu Province, Institute of Chemical Industry of Forest Products, Chinese Academy of Forestry, Nanjing, Jiangsu 210042, China*

*^3^ College of Chemical Engineering, Nanjing Forestry University, Nanjing, Jiangsu 210042, China*

Correspondence should be addressed to Jianchun Jiang, jiangjc@icifp.cn; Mengmeng Fan, fanmengmeng370@njfu.edu.cn and Kui Wang, wangkui@caf.ac.cn


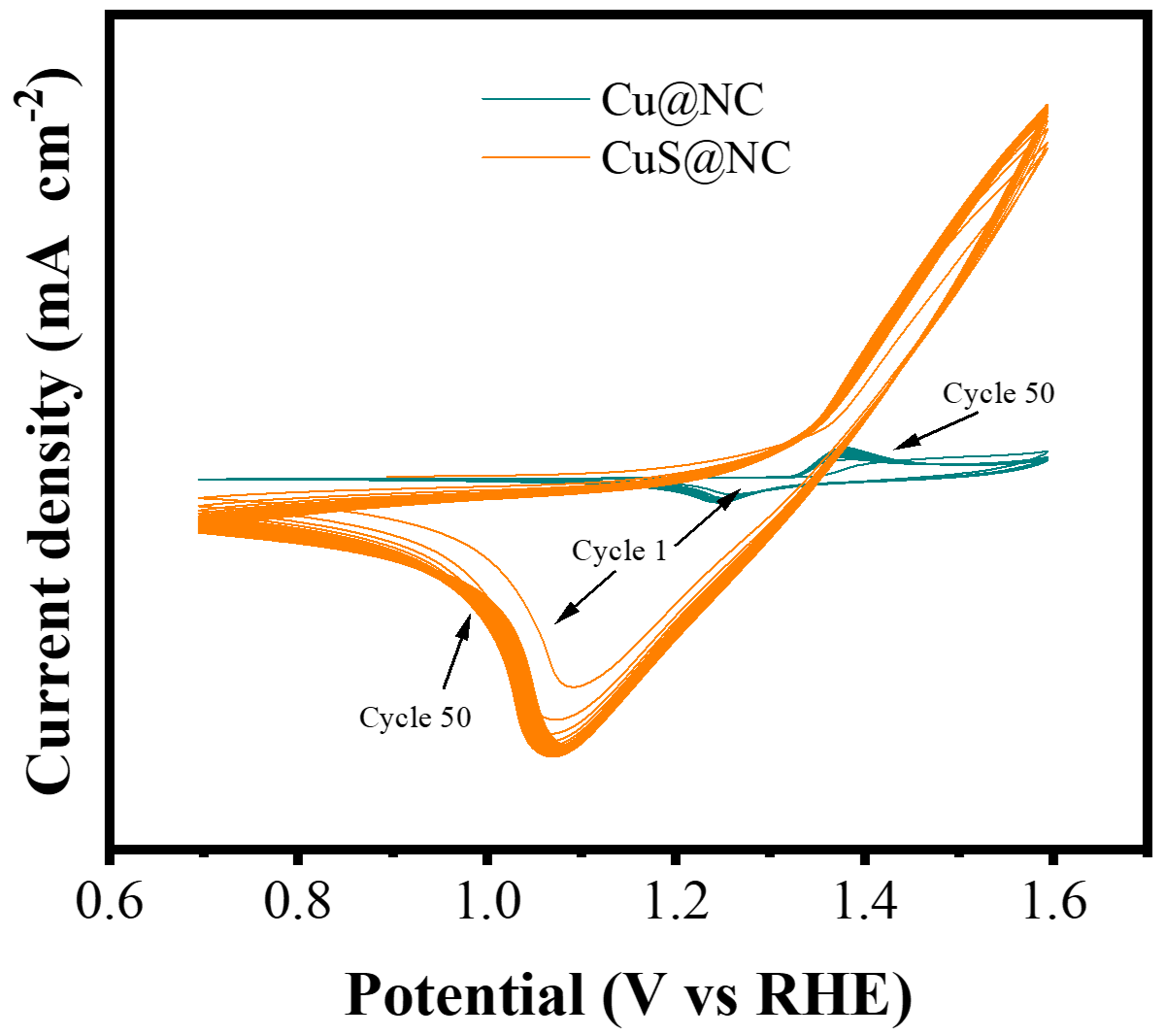


**Fig. S11** The CV curves for Cu@NC and CuS@NC electrode activation.


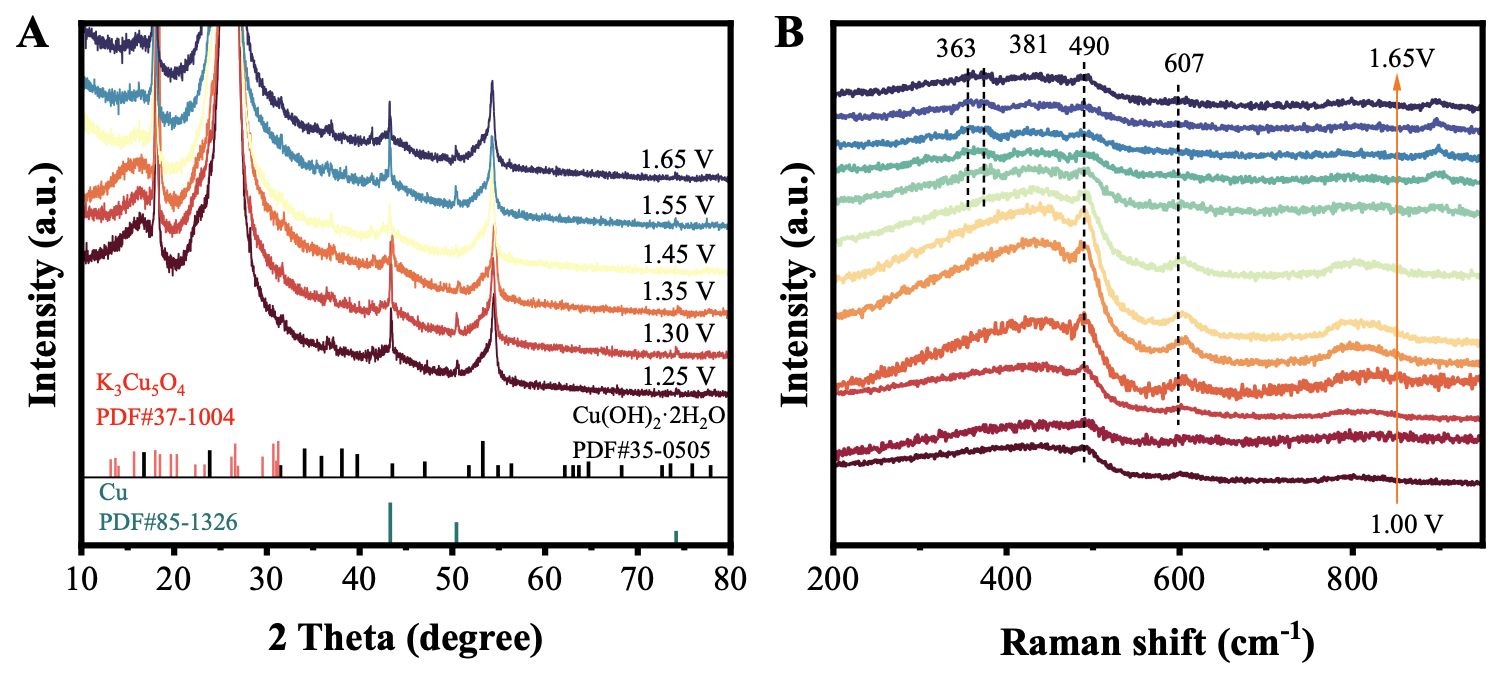


**Fig. S12** The quasi-in-situ XRD (A) and in-situ Raman (B) spectra of Cu@NC.


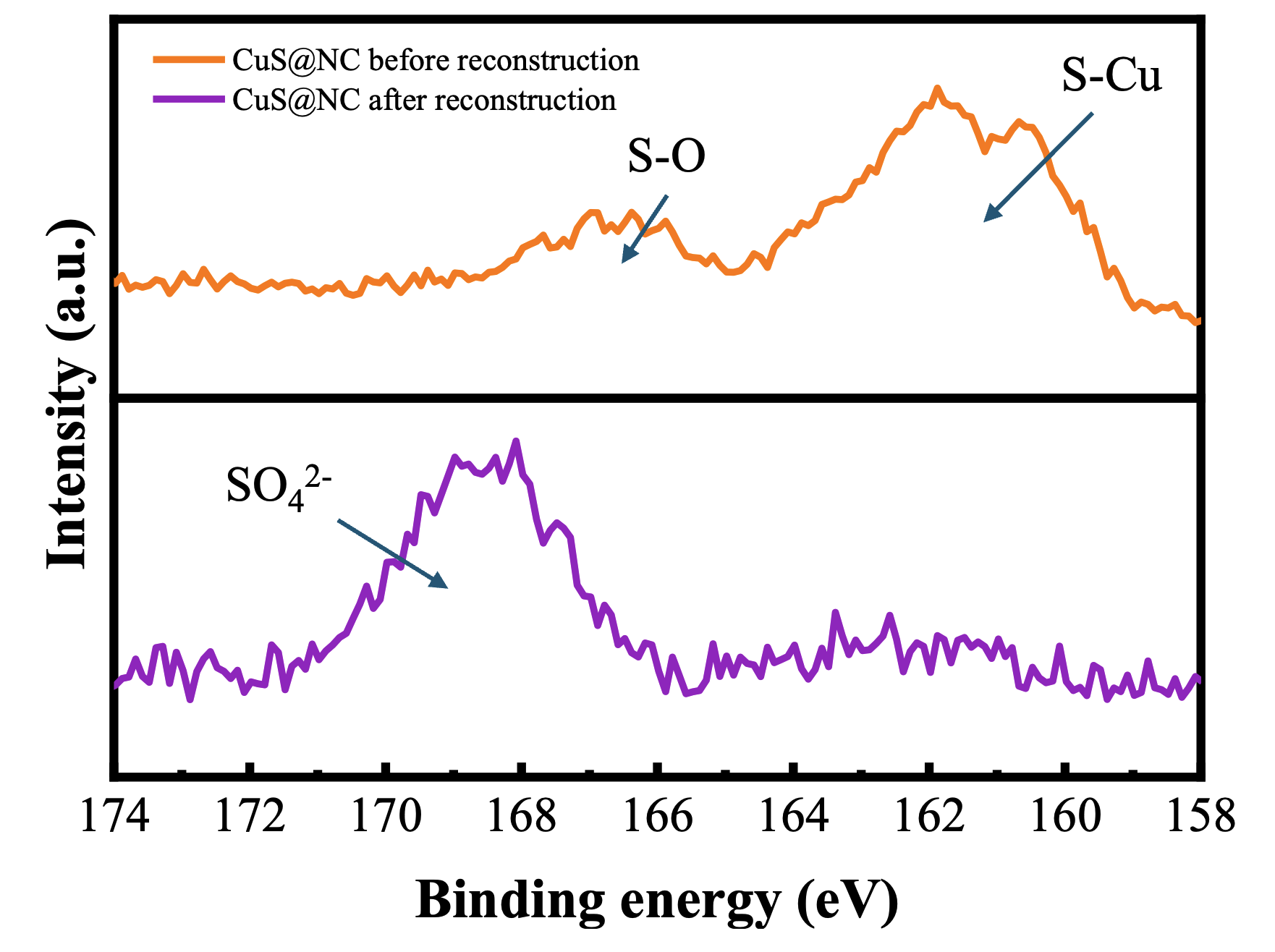


**Fig. S13** S 2p spectrum of CuS@NC electrode before and after reconstruction.


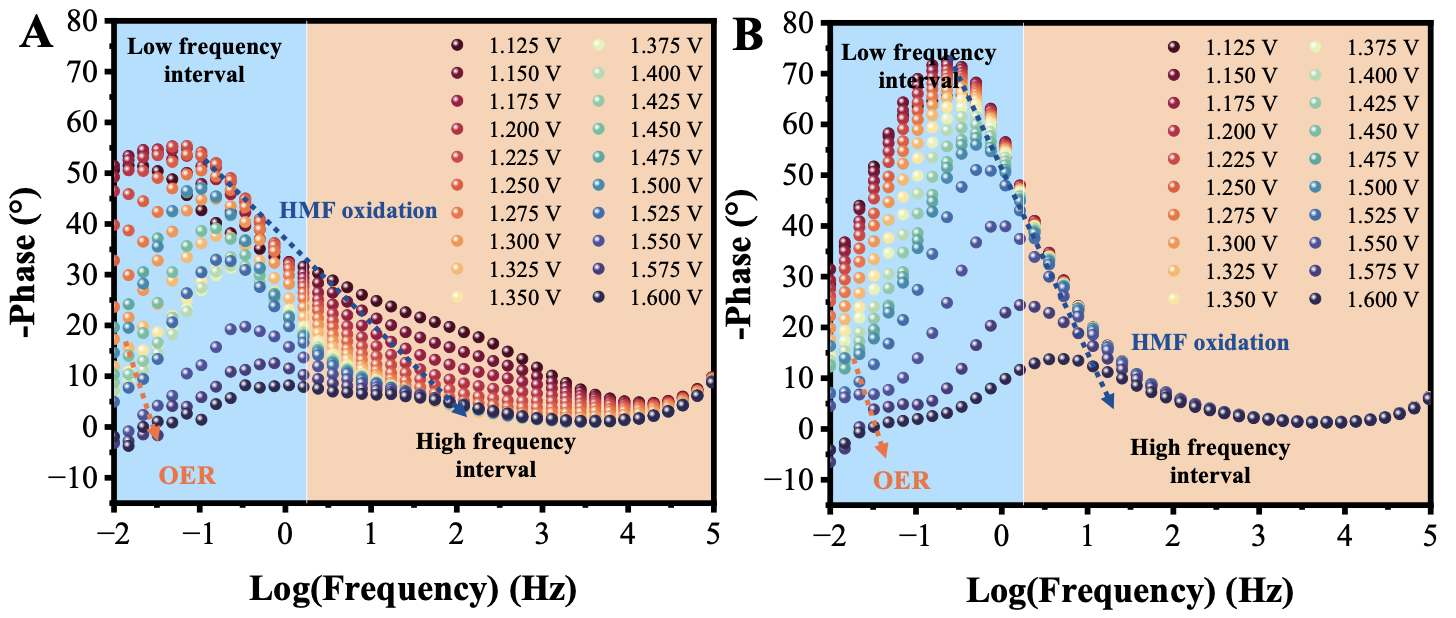


**Fig. S14** Bode phase plots of Cu@NC (A) and CuS@NC (B) in 1 M KOH + 100 mM HMF.


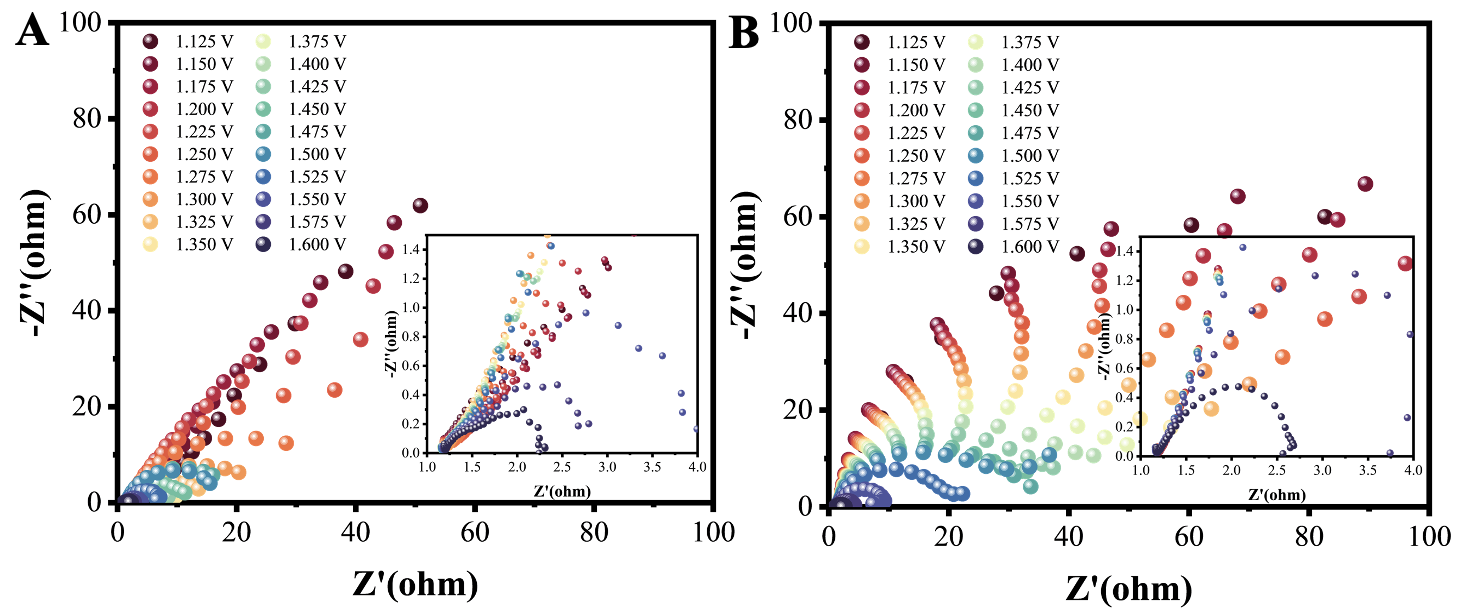


**Fig. S15** Nyquist plots of Cu@NC (A) and CuS@NC (B) in 1 M KOH + 100 mM HMF.


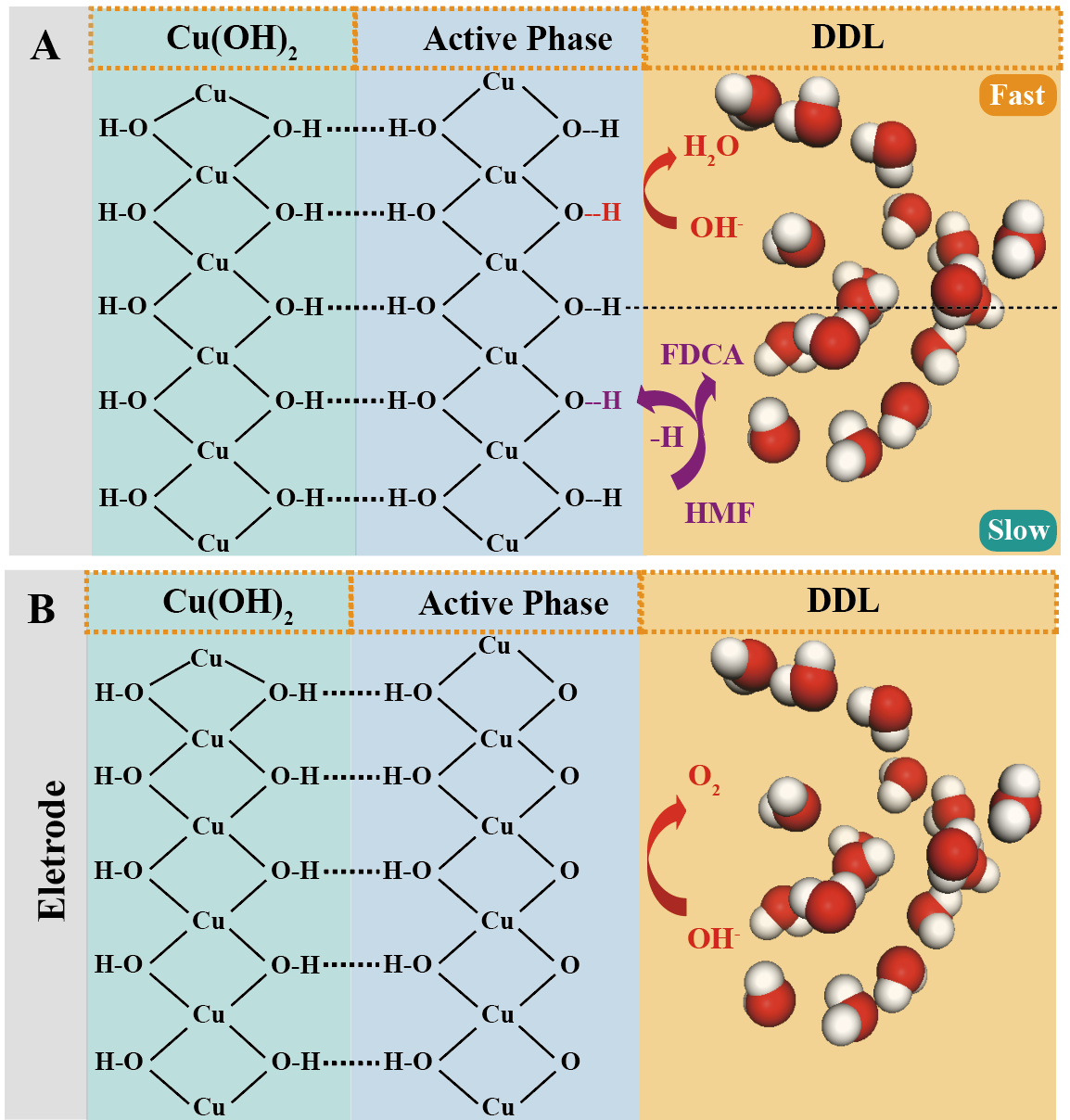


**Fig. S16** Schematic illustrations of HMFOR (A) and OER (B) system at reconstructed Cu@NC electrode interface (Cu(OH)_2_).


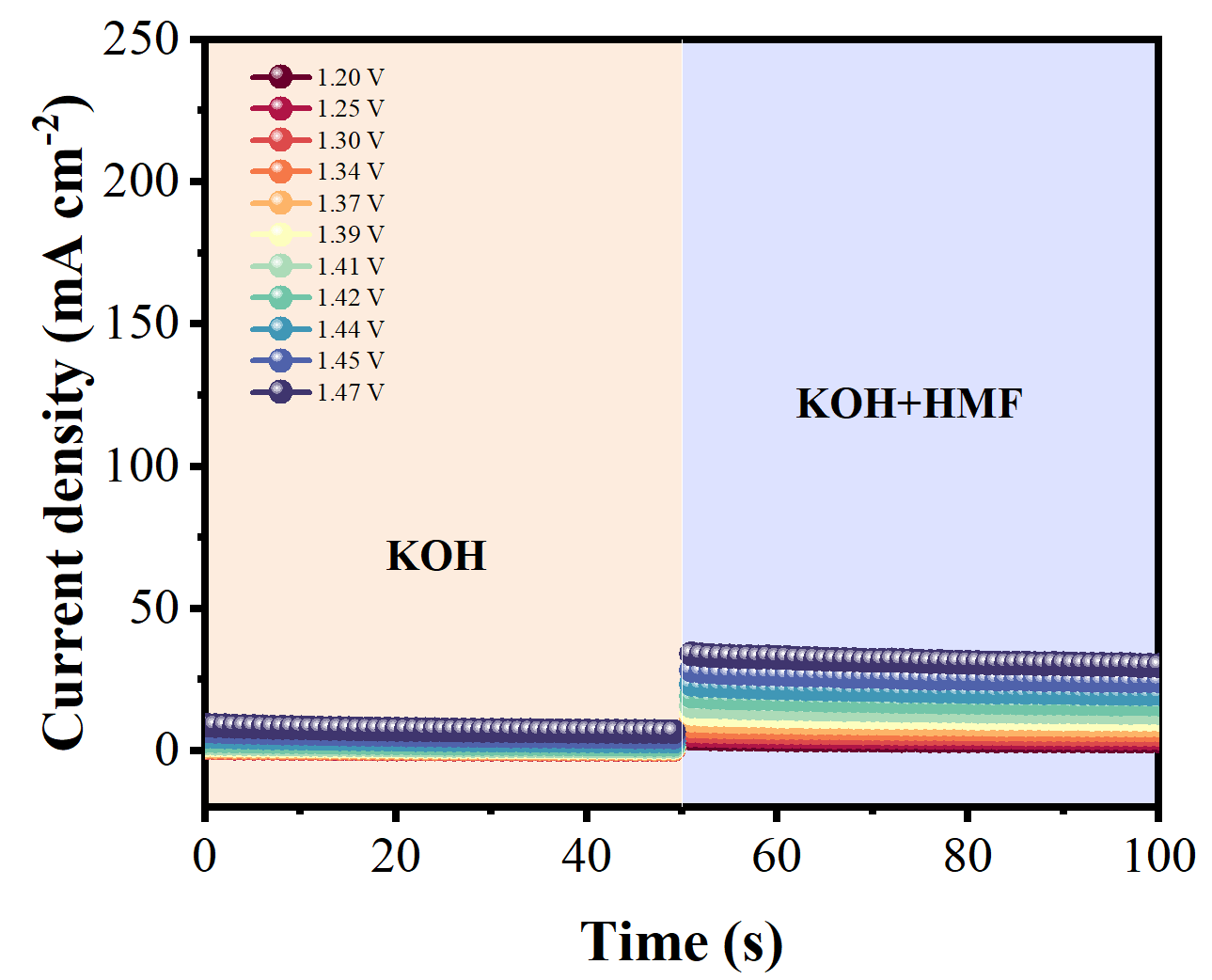


**Fig. S17** The i-t curve of Cu@NC in KOH and KOH with 100 mM HMF.


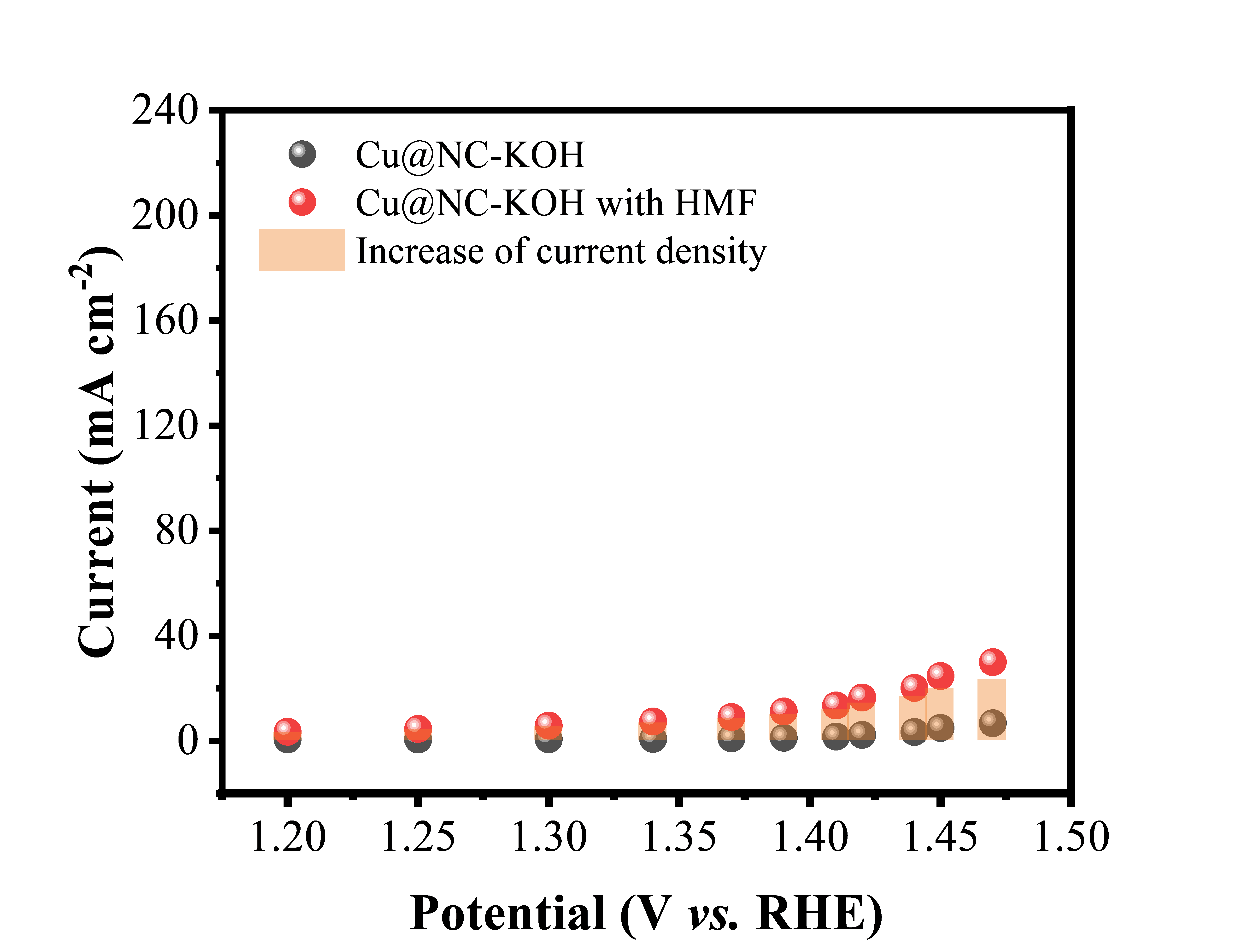


**Fig. S18** The current density from the i-t curves at different potentials of Cu@NC.


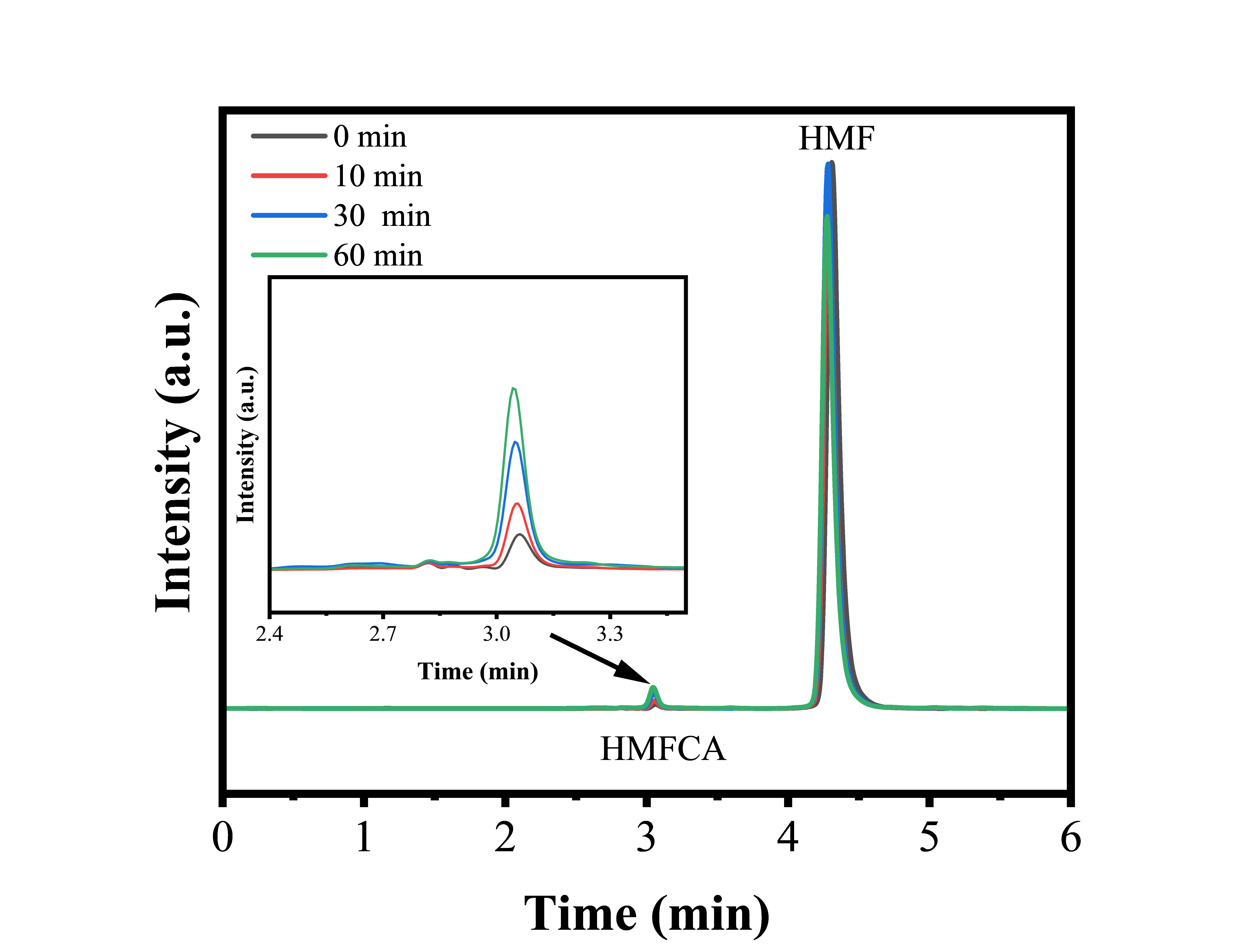


**Fig. S19** HPLC test results for the spontaneous oxidation of 100 mM HMF in 1 M KOH.


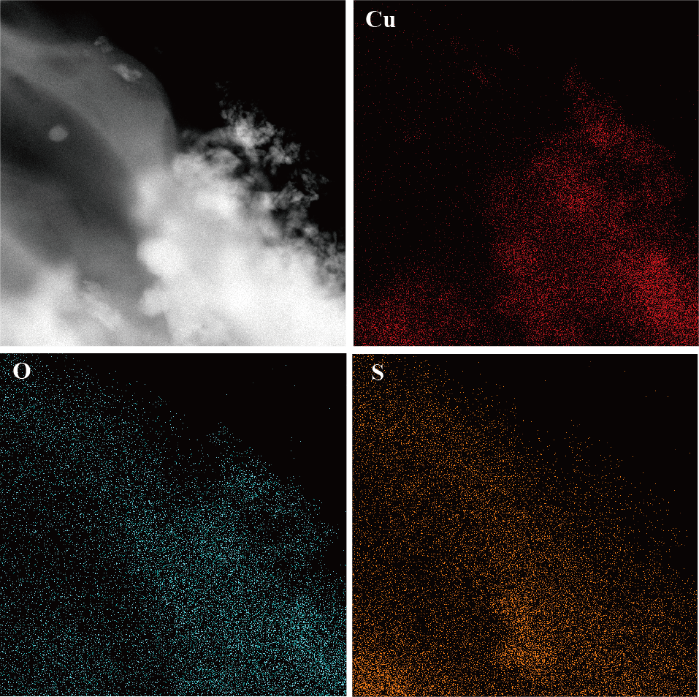


**Fig. S20** TEM-mapping image of CuS@NC after cycling tests.
