# Supplementary material for: S-Species-Stimulated Deep Reconstruction of Ultra-Homogeneous CuS Nanosheets for Efficient HMF Electrooxidation
Source: Research (Wash D C). 2025 Nov 14;8:0925. doi: 10.34133/research.0925 (PMC12615154; doi:10.34133/research.0925)
Supplement: Supplementary 1 — Figs. S1 to S20 Scheme S1 Tables S1 to S3 Movies S1 and S2 [file research.0925.f1.zip › SUPPLEMENTARY MATERIALS_Tables S1 to S3.docx]

**S-species****-Stimulated Deep-reconstruction of Ultra-homogeneous CuS Nanosheets for Efficiently HMF Electrooxidation**

Yongzhi Xiong ^1^, Mengyuan Qiu ^1^, Yihan Wang ^1^, Qi Liu^1^, Dong Ouyang ^1^, Yajun Liu ^1^, Changzhou Chen ^1^, Jianchun Jiang ^1, 2, *^, Mengmeng Fan ^3,*^, Kui Wang ^1, 2, *^

*^1^ Institute of Advanced Carbon Conversion Technology, Fujian Provincial Key Laboratory of Biomass Low-Carbon Conversion, Huaqiao University, Xiamen, Fujian 361021, China*

*^2^ Key Lab. of Biomass Energy and Material of Jiangsu Province, Institute of Chemical Industry of Forest Products, Chinese Academy of Forestry, Nanjing, Jiangsu 210042, China*

*^3^ College of Chemical Engineering, Nanjing Forestry University, Nanjing, Jiangsu 210042, China*

Correspondence should be addressed to Jianchun Jiang, jiangjc@icifp.cn; Mengmeng Fan, fanmengmeng370@njfu.edu.cn and Kui Wang, wangkui@caf.ac.cn

**Table S1** DFT reaction energy

| **Number of Steps** | **Name of steps** | **ΔE/eV** | |
| --- | --- | --- | --- |
|  |  | **Cu(OH)_2_** | **SO_4_-Cu(OH)_2_** |
| 1→2 | Adsorption energy of HMF | 0.23 | -0.04 |
| 2→3 | Dehydrogenation of Cu-O-H | 1.51 | 0.89 |
| 3→4 | Dehydrogenation of α-C-H | -0.99 | -0.20 |
| 4→5 | Formation of  -COOH | -0.60 | -0.89 |
| 5→6 | Dehydrogenation of Cu-O-H | 1.03 | 0.67 |
| 6→7 | Dehydrogenation of α-C-H | -0.98 | -0.57 |
| 7→8 | Dehydrogenation of Cu-O-H | 1.48 | 0.93 |
| 8→9 | Dehydrogenation of -O-H | -0.70 | 0.11 |
| 9→10 | Dehydrogenation of Cu-O-H | 1.20 | 0.61 |
| 10→11 | Dehydrogenation of α-C-H | -0.21 | 0.26 |
| 11→12 | Formation of  -COOH | -1.24 | -0.96 |
| 12→13 | Desorption energy of FDCA | 0.15 | 0.12 |

**Table S2** Bader charge

| \ | | | Co(OH)_2_ | | | TOP-SO_4_\|Cu(OH)_2_ | | | |
| --- | --- | --- | --- | --- | --- | --- | --- | --- | --- |
| Functional groups | Atoms | Number of e^-^ in pseudopotential | | Bader Charge | Charge transfer | | Bader Charge | Charge transfer |  |
| -CHO | C(-C-H/-C=O) | 4 | | 3.14 | -0.86 | | 3.11 | -0.89 |  |
|  | H(-C-H) | 1 | | 0.97 | -0.03 | | 0.95 | -0.05 |  |
|  | O(-C=O) | 6 | | 7.07 | 1.07 | | 7.13 | 1.13 |  |
| -CH_2_OH | C(-C-H/-C-O) | 4 | | 3.61 | -0.39 | | 3.47 | -0.53 |  |
|  | -H(-C-H-1) | 1 | | 0.84 | -0.16 | | 0.97 | -0.03 |  |
|  | -H(-C-H-2) | 1 | | 0.93 | -0.07 | | 0.91 | -0.09 |  |
|  | -H(-O-H) | 1 | | 0.39 | -0.61 | | 0.35 | -0.65 |  |
|  | O(-O-H) | 6 | | 7.08 | 1.08 | | 7.13 | 1.13 |  |

**Table S3** Model Schottky fitting parameters

| **Catalysts** | **f/Hz** | **k** | **NA/cm^-3^** | **E_fb_/V vs RHE** |
| --- | --- | --- | --- | --- |
| Cu@NC | 1*10^3^ | -6.24*10^7^ | 1.95*10^23^ | 1.45 |
|  | 3*10^3^ | -1.69*10^8^ | 7.21*10^22^ | 1.40 |
|  | 5*10^3^ | -5.96*10^8^ | 2.05*10^22^ | 1.34 |
| CuS@NC | 1*10^3^ | 1.12E*10^7^ | 1.09*10^24^ | 0.72 |
|  | 3*10^3^ | -6.80*10^6^ | 1.79*10^24^ | 1.57 |
|  | 5*10^3^ | -1.58E*10^7^ | 7.70*10^23^ | 1.50 |
